# Catalyst-Free Spontaneous Aza-Mannich/Lactamization Cascade Reaction: Easy Access to Polycyclic δ-Lactams

**DOI:** 10.3390/molecules30132702

**Published:** 2025-06-23

**Authors:** Antonia Di Mola, Caterina Vietri, Consiglia Tedesco, Antonio Massa

**Affiliations:** Dipartimento di Chimica e Biologia “A. Zambelli”, Università degli Studi di Salerno, Via Giovanni Paolo II, 84084 Fisciano, Italy; c.vietri12@studenti.unisa.it (C.V.); ctedesco@unisa.it (C.T.)

**Keywords:** polycyclic compounds, heterocycles, isoquinoline, cascade reaction

## Abstract

Ring-fused azacyclic compounds are important building blocks in the synthesis of natural products and pharmaceutical agents. Herein, we report an effective and valuable one-pot approach to obtaining polycyclic fused δ-lactams from readily available 2-formylphenyl acetate and diamines under catalyst-free and green conditions.

## 1. Introduction

**Figure 1:**
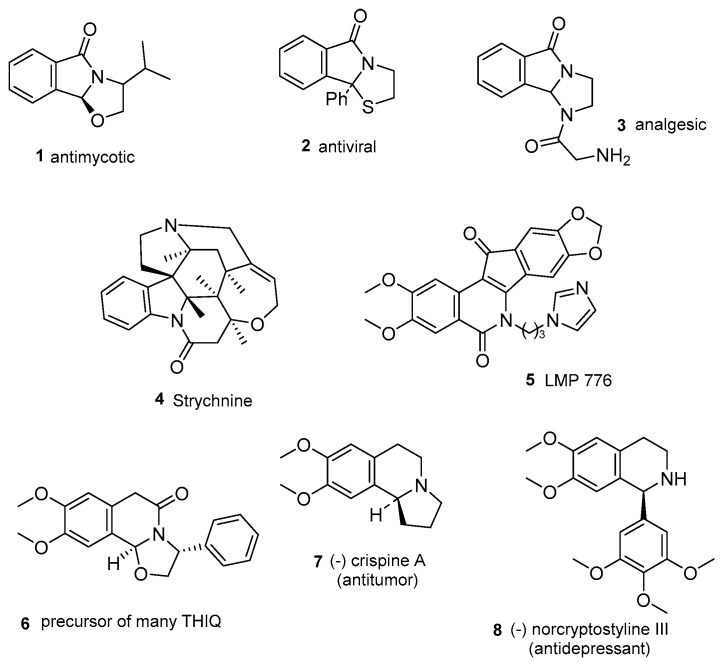
Examples of polycyclic aza-lactams.

Polycyclic compounds, which contain multiple fused rings in their structure, are important scaffolds in the development of several drugs [1,2]. In particular, polycyclic aza-lactams are of large interest in organic and medicinal chemistry due to their wide range of biological activities. For example, γ-lactam **1** has activity against *Candida albicans* [3]; γ-lactam **2** shows non-nucleoside HIV reverse transcriptase inhibition [4,5]; and γ-lactam **3** possesses anti-inflammatory, analgesic, tranquilizing, and antitussive properties [6]. Also, polycyclic δ-lactams are fascinating compounds: strychnine **4** is a toxic alkaloid historically used to strengthen muscle contractions [7], while LMP776 has undergone clinical trials for the treatment of solid cancer [8]. In addition, polycyclic δ-lactams offer the possibility to be easily transformed into related heterocycles, as reported by Amat in 2010 [9]. In fact, the polycyclic lactam **5** is a key intermediate in the synthesis of 1-substituted tetrahydroisoquinoline alkaloids (THIQ; see Figure 1), a moiety found in many bioactive natural products such as **6**–**8** [10,11,12].

Cascade reactions are a powerful approach for the construction of carbon and heterocyclic ring structures. They respect many paradigms of green chemistry like step, pot, and atom economy [13,14]. However, most of the reported methods for the construction of polycyclic lactams are based on transition-metal catalysis [15,16] and/or use halogenated or aromatic solvents [17,18]. Consequently, developing novel and efficient transformations to synthesize some important heterocyclic compounds under transition-metal-free reaction conditions is still highly desirable.

In the present investigation, continuing our program aimed at preparing valuable heterocyclic compounds by diverse cascade and multicomponent reactions [19,20,21,22], we envisaged the possibility of developing a convenient route to obtain polycyclic δ-lactams, focusing on the bifunctional aromatic compound 2-formylphenylacetate **9a**.

**Scheme 1:**
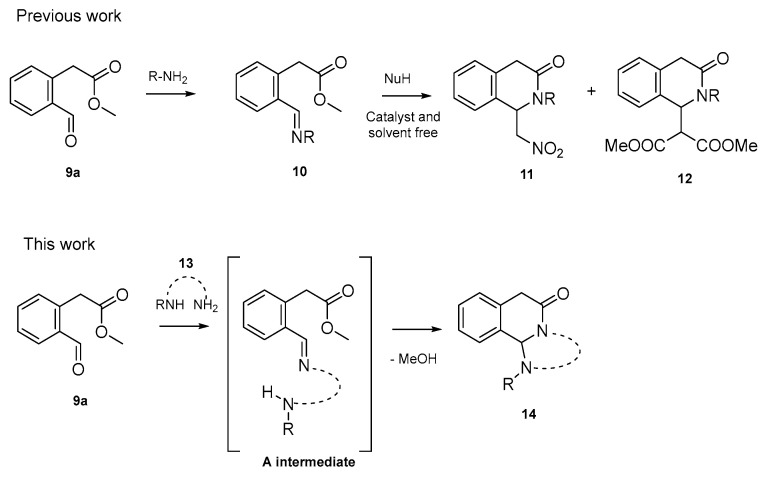
Previous work and present investigation.

Successful cascade reactions rely on the careful design of suitable starting materials capable of undergoing sequential, hierarchical transformations. In recent years, our attention has focused on bis-electrophilic compounds, such as 2-cyanobenzaldehydes [19,20] or α-amido sulfones derived from 2-formylbenzoate [21,22]. These systems feature one electrophilic group that is more reactive, enabling the formation of a high-energy intermediate. This intermediate can then undergo intramolecular cyclization with the second, less reactive electrophilic group, facilitating efficient and selective ring closure. In this context, 2-formylphenyl acetate **9a** has proven particularly effective for the synthesis of 3-isoquinolones, a class of δ-lactams, via a Mannich-initiated cascade reaction. This transformation involves imines derived from **9a**, nitromethane, and dimethyl malonate under catalyst- and solvent-free conditions (Scheme 1) [23]. 

Furthermore, 2-formylphenyl acetates have been utilized in the synthesis of 3-isoquinolones by Lewis acid-catalyzed domino Strecker-lactamization [24], in Ni(II)-catalyzed reactions [25], in the presence of Grignard reagents [26], and in cyclo-condensation with (*R*)-phenylglycinol [8]. However, to the best of our knowledge, there are no examples in the literature of the reaction between **9** and diamine **13**. According to the proposed mechanistic hypothesis (Scheme 1), a primary amine group of suitable diamines should react with the aldehyde group of **9** to give the imine intermediate **A**. Then, the second amino group should intramolecularly attack the very reactive imine group to form a cyclic aminal intermediate, which can undergo lactamization to afford new polycyclic products such as **14**.

## 2. Results

Based on the mechanistic hypothesis depicted in Scheme 1, the reaction between **9a** and 1,3-diaminopropane **13a**, chosen as the diamine model, was investigated in the green solvent ethanol, without the use of any catalyst or additive (Table 1). To find the best conditions for obtaining these valuable heterocycles, we examined not only the reaction parameters, such as the **9a** concentration and reaction time, but also the purification procedure. The evidence that purification can also affect the isolated yield was highlighted by an ^1^H NMR analysis of the crude, which revealed good conversions, but the isolated yields were relatively low (compare Entries 1 and 2 with Entries 3 and 4). The reaction was spontaneous in ethanol, which was used as solvent, but the amount of isolated product increased as the stationary phase used for purification was changed from silica gel to alumina. When using silica or silica deactivated with 1% Et_3_N, only moderate yields were obtained. This outcome was probably due to the stronger interaction between the silica OH groups and the amino/amide groups (Entries 1 and 2). Indeed, when we moved to alumina, better results were achieved (Entries 3 and 4). Then, with more reliable purification in hand, the reaction conditions were optimized to achieve quantitative conversions by performing the reaction for a longer time and with a higher molar concentration of 0.56 mol/L (Entry 5). Other conditions were also investigated, such as more diluted concentrations (Entries 6 and 7) and an increased reaction temperature of 60 °C (Entry 8); however, these resulted in lower efficiency.

**Figure 2:**
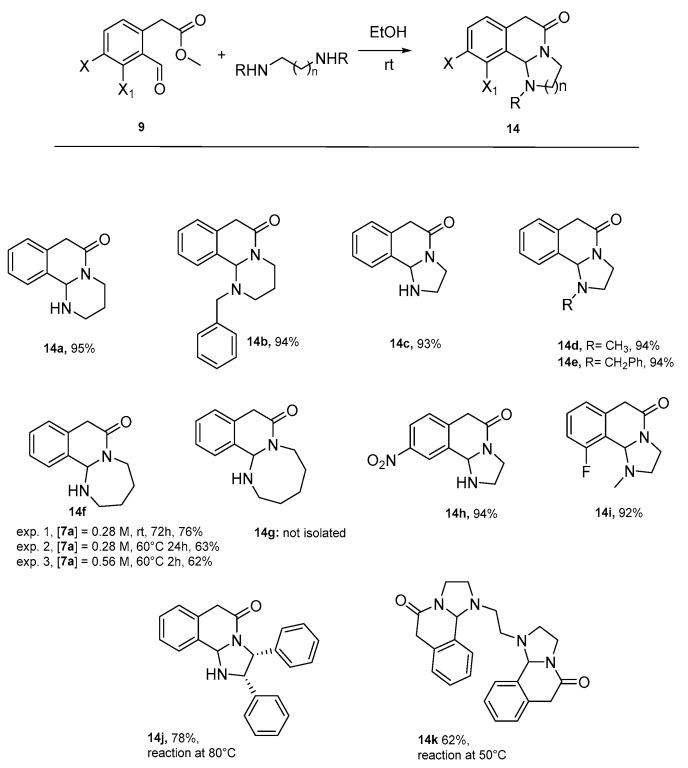
Analysis of the scope of the aza-Mannich/lactamization process.

**Figure 3:**
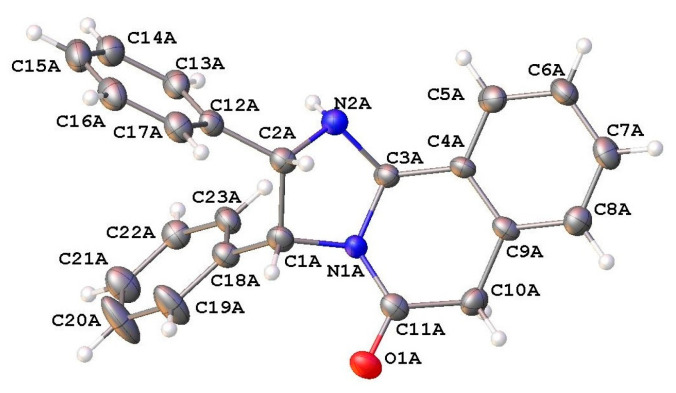
ORTEP drawing of compound **14j**. Ellipsoids are shown at 50% probability level.

With the optimized conditions in hand, the scope of the reaction was then analyzed using substituted 2-formylphenyl acetates **9** and different diamines **13**. In most cases, we obtained the expected new products in high yields, irrespective of the presence of fluorine atoms or nitro electron-withdrawing groups on the aromatic ring of 2-formylphenylacetate (Figure 2). When 1,4-diaminobutane was used, the tricycle compound **14f** containing a 1,3-diazepino seven-membered ring was obtained in 76% yield after 72 h under more diluted conditions. Other conditions, such as variations in the concentration reaction temperature and reaction time, were also tested, but lower efficiency was observed (Figure 2). On the other hand, no product was observed when using 1,5-diaminopentane, even when heating up to 80 °C. This outcome was not surprising since the construction of eight-membered rings is a common challenge in chemistry, often due to the strain and unfavorable kinetics of forming such rings [27]. Also, the presence of secondary amines, such as *N*-methylethylenadiamine, *N*-benzylethylendiamine, or *N*-benzyl-1,3-diaminopropane (**14b**, **d**, **e**, **i**), or a hindered diamine, like 1,2-diphenylethylamine, enabled the isolation of tricyclic products in good yields. In the latter case, only one diastereomer was obtained, and the structure of **14j** was confirmed by an X-ray diffraction analysis (Figure 3), showing a 1,3-trans stereochemistry [28]. Interestingly, when using triethylenetetramine, the dimeric structure **14k** was obtained in 62% yield as a mixture of two diastereomers. The reaction also performed well in the presence of other 2-formylphenyl acetates further substituted on the aromatic ring (**14h** and **14i**).

**Figure 4:**
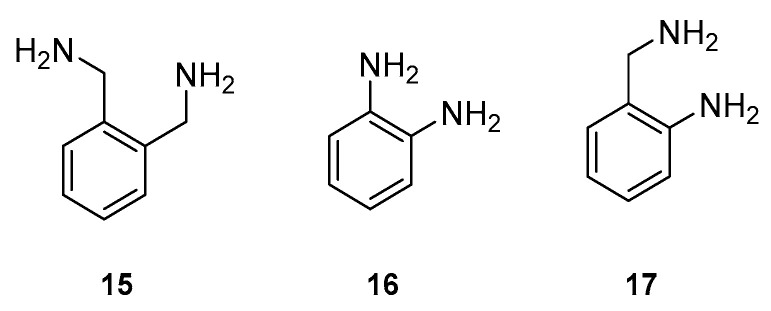
Structures of other amines investigated.

**Figure 5:**
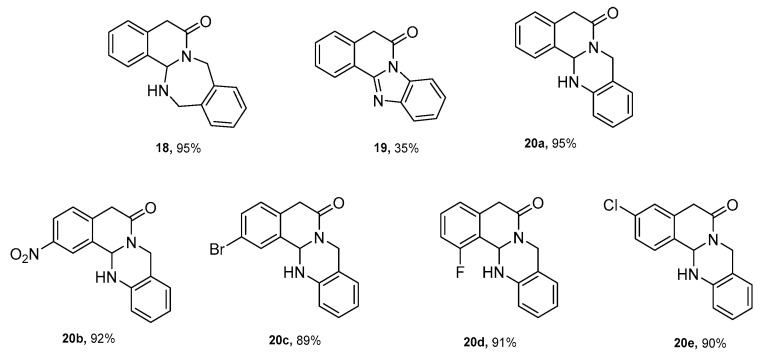
Tetracyclic δ-lactam scope.

To further enlarge the scope of the reaction with the aim of preparing tetracyclic δ-lactams, other readily available or easily preparable aromatic diamines were used in the reaction with aldehyde **9**, such as *o*-xylilendiamine **15**, *o*-phenylendiamine **16**, and 2-aminobenzylamine **17** (Figure 4 and Figure 5).

As reported in Figure 5, the 1,3-diazepino polycyclic lactam **18** was obtained in high yield using diamine **15**. When *o*-phenylendiamine **16** was used, the corresponding oxidized product **19**, already known in the literature [29], was obtained, though in a rather low yield due to the formation of decomposition products. The analysis of the scope focused on the use of the asymmetric diamine **17** and was extended to other 2-formylphenyl acetates further substituted on the aromatic ring in different positions, leading to the desired products **20a**–**20e** in high yields and regioselectivity.

**Scheme 2:**
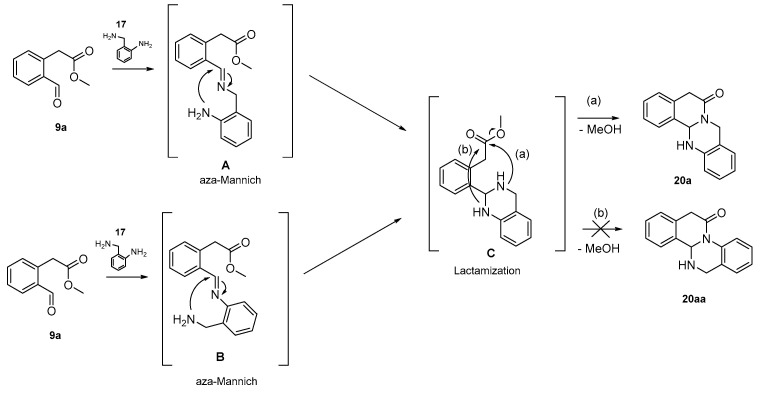
Reactivity of 2-formylphenylacetate **9a** in the presence of 2-aminobenzylamine **17**.

The preparation of tetracyclic δ-lactams derived from 2-aminobenzylamine **17** could pose concerns about the regioselectivity, according to the mechanism reported in Scheme 2. In the reaction, only one regioisomer was obtained, as detected by an ^1^H NMR of the crudes. Analyzing the reaction mechanism in more detail, the spontaneous cascade process should consist of imine formation, intramolecular aza-Mannich attack, and final lactamization. We suppose that the observed regioselectivity depends on the higher nucleophilicity of the aliphatic NH group with respect to the NH anilino group of intermediate **C** (pathway a), leading to only the regioisomer **20a**.

**Scheme 3:**
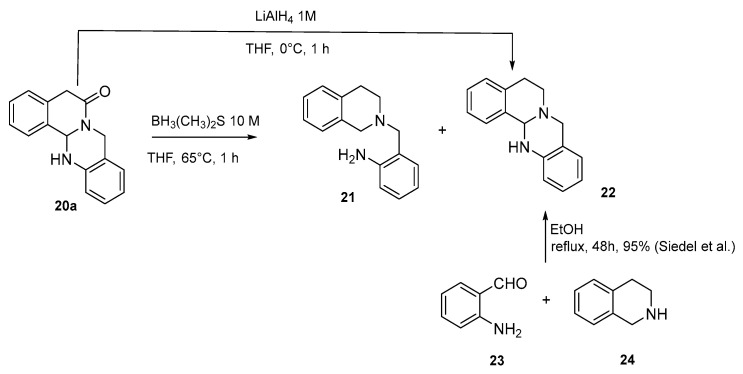
Second reactivity of compound **20a** [30].

Under the optimized conditions reported (Table 1, Entry 5), the tetracyclic δ-lactam **20a** was recovered in a short reaction time and in very high yields. Since attempts to obtain suitable crystals for X-rays failed, to assess the structure of the obtained compound **20a**, we investigated its second reactivity, such as the amide reduction (Scheme 3), and compared our spectra with those reported in the literature [30,31]. In fact, quinazoline **22** is an already known compound prepared by the reaction of 2-aminobenzaldeyde **23** and 1,2,3,4-tetrahydroisoquinoline **24** in refluxing ethanol for 48 h [30]. The treatment of **20a** with BH_3_(CH_3_)_2_S allowed us to recover compound **22** only in traces because the main product was the tetrahydroisoquinoline **21** (94% yield) in which the amide group was reduced and the aminal bond was cleaved. Notably, compound **21**, previously obtained by a different methodology, belongs to a class of bioactive α_2_-adrenergic receptor antagonists [32]. Indeed, when **20a** was treated with LiAlH_4_ 1 M solution in THF, **22** was obtained in a very good yield (89%), confirming that pathway (a) is more plausible, as reported in Scheme 2.

## 3. Materials and Methods

Unless otherwise noted, all chemicals, reagents, and solvents for the performed reactions were commercially available. Aldehydes were prepared according to the literature procedures [15]. All the reactions were monitored by thin-layer chromatography (TLC) on precoated silica gel plates (0.25 mm) and visualized by fluorescence quenching at 254 nm. Flash chromatography was carried out using neutral activated alumina (Merck, Darmstadt, Germany). Yields are given for isolated products showing one spot on a TLC plate. The NMR spectra were recorded on Bruker DRX 600, 400, 300, and 250 MHz spectrometers (600 MHz, ^1^H, 150 MHz, ^13^C; 400 MHz, ^1^H, 100.6 MHz; ^13^C, 300 MHz, ^1^H, 75.5 MHz, ^13^C, 250 MHz, ^1^H, 62.5 MHz, ^13^C). The internal reference was set to the residual solvent signals (δ_H_ 7.26 ppm, δ_C_ 77.16 ppm for CDCl_3,_ δ_H_ 2.50 ppm, δ_C_ 39.10 ppm for DMSO-*d*_6_). The ^13^C NMR spectra were recorded under broad-band proton decoupling. ^1^HNMR data and HRMS are reported for all compounds. IR and ^13^CNMR data are given only for unknown compounds. The following abbreviations are used to indicate the multiplicity in the NMR spectra: s—singlet, d—doublet, t—triplet, q—quartet, dd—doublet of doublets, m—multiplet, and br s—broad signal. High-resolution mass spectra (HRMS) were acquired using a Bruker SolariX XR Fourier transform ion cyclotron resonance mass spectrometer (Bruker Daltonik GmbH, Bremen, Germany) equipped with a 7T refrigerated actively shielded superconducting magnet. For ionization of the samples, electrospray ionization (ESI) or MALDI was applied. IR spectra were recorded on a IR Bruker Vertex 70v spectrometer.

### 3.1. General Procedure for the Synthesis of Tricyclic Lactams ***14a***–***14k***

To a solution of aldehyde **9** (50 mg, 0.28 mmol) in EtOH (500 µL), diamine **13** (0.28 mmol, 1 eq.) was added, and the mixture was stirred at room temperature for 24 h. The solvent was evaporated, and the crude was purified on a short column of neutral alumina (from Pentane 20% in CH_2_Cl_2_ to CH_2_Cl_2_).

***1,2,3,4,7,11b-hexahydro-6H-pyrimido [2,1-a]isoquinolin-6-one 14a***

Yellow solid. Yield: 54 mg, 95%. M.p. 147 °C (dec.). Rf. 0.30 (DCM 100/MeOH 1, SiO_2_). ^1^H NMR (400 MHz, CDCl_3_): δ 7.46 (d, *J* = 7.6 Hz, 1H), 7.27–7.25 (m, 2H), 7.09 (d, *J* = 7.6 Hz, 1H), 5.29 (s, 1H), 4.97 (dd, *J*_1_ = 12.8 Hz, *J*_2_ = 2.0 Hz, 1H), 3.73 (d, *J* = 21.6 Hz, 1H), 3.65 (d, *J* = 21.6 Hz, 1H), 3.30 (dd, *J*_1_ = 13.6 Hz, *J*_2_ = 2.0 Hz, 1H), 3.16–3.08 (m, 1H), 2.90–2.83 (m, 1H), 1.94 (br s, 1H, NH), 1.68–1.63 (m, 2H). ^13^CNMR (100 MHz, CDCl_3_): δ 166.4, 131.7, 130.8, 128.5, 127.3, 127.2, 126.8, 73.2, 45.8, 42.3, 34.8, 26.9. HRMS (MALDI-FT ICR): *m*/*z* calcd for [C_12_H_14_N_2_O + H]^+^: 203.1179; found: 203.1179. IR (KBr): 3230, 1717, 689, 650 cm^−1^.

***1-benzyl-1,2,3,4,7,11b-hexahydro-6H-pyrimido [2,1-a]isoquinolin-6-one 14b***

Slightly brown solid. Yield: 76 mg, 94%. M.p. 178 °C (dec.). Rf. 0.50 (DCM 100/MeOH 1, SiO_2_). ^1^H NMR (400 MHz, CDCl_3_): δ 7.46 (d, *J* = 8.0 Hz, 1H), 7.30–7.09 (m, 8H), 5.62 (s, 1H), 4.99 (dd, *J*_1_ = 13.2 Hz, *J*_2_ = 4.8 Hz, 1H), 3.78 (d, *J* = 21.2 Hz, 1H), 3.68 (d, *J* = 21.2 Hz, 1H), 3.47 (d, *J* = 13.2 Hz, 1H), 3.32 (d, *J* = 13.2 Hz, 1H), 3.10–2.99 (m, 1H), 2.90 (dt, *J*_1_ = 13.2 Hz, *J*_2_ = 3.2 Hz, 1H), 2.21–2.08 (m, 1H), 1.32–1.25 (m, 2H). ^13^CNMR (100 MHz, CDCl_3_): δ 167.4, 138.8, 132.3, 130.7, 128.6 (2C), 128.5, 128.3 (2C), 127.4, 127.3, 127.1, 127.0, 78.1, 48.3, 47.8, 42.7, 35.0, 18.9. HRMS (MALDI-FT ICR): *m*/*z* calcd for [C_19_H_20_N_2_O + H]^+^: 293.1648; found: 293.1645. IR (KBr): 1717, 687, 647 cm^−1^.

***2,3,6,10b-tetrahydroimidazo [2,1-a]isoquinolin-5(1H)-one 14c***

Yellow solid. Yield: 49 mg, 93%. M.p. 91 °C (dec.). Rf. 0.20 (DCM 100/MeOH 1, SiO_2_). ^1^H NMR (400 MHz, CDCl_3_): δ 7.51–7.49 (m, 1H), 7.31–7.29 (m, 2H), 7.18 (d, *J* = 6.5 Hz, 1H), 5.33(s, 1H), 3.72–3.45 (m, 5H), 3.30–3.23 (m, 1H), 2.72 (br s, 1H). ^13^CNMR (75 MHz, CDCl_3_): δ 165.9, 133.2, 132.0, 128.9, 127.6, 127.3, 124.4, 74.4, 45.2, 44.3, 34.8. HRMS (MALDI-FT ICR): *m*/*z* calcd for [C_11_H_12_N_2_O + H]^+^: 189.1022; found: 189.1024. IR (KBr): 3280, 1711, 690, 652 cm^−1^.

***1-methyl-2,3,6,10b-tetrahydroimidazo [2,1-a]isoquinolin-5(1H)-one 14d***

Yellow solid. Yield: 55 mg, 94%. M.p. 77 °C (dec.). Rf. 0.30 (DCM 100/MeOH 1, SiO_2_). ^1^H NMR (400 MHz, CDCl_3_): δ 7.47–7.44 (m, 1H), 7.31–7.28 (m, 2H), 7.21–7.18 (m, 1H), 5.00 (s, 1H), 3.70–3.54 (m, 4H), 3.28 (dt, *J*_1_ = 7.2 Hz, *J*_2_ = 2.4 Hz, 1H), 3.06–2.99 (m, 1H), 2.42 (s, 3H). ^13^C NMR (100 MHz, CDCl_3_): δ 167.1, 133.3, 132.5, 128.3, 127.6, 126.9, 124.4, 79.0, 54.2, 41.6, 39.9, 38.3. HRMS (MALDI-FT ICR): *m*/*z* calcd for [C_12_H_14_N_2_O + H]^+^: 203.1179; found: 203.1181. IR (KBr): 1716, 689, 653 cm^−1^.

***1-benzyl-2,3,6,10b-tetrahydroimidazo [2,1-a]isoquinolin-5(1H)-one 14e***

Yellow oil. Yield: 73 mg, 94%. Rf. 0.45 (DCM 100/MeOH 1, SiO_2_). ^1^H NMR (400 MHz, CDCl_3_): δ 7.60–7.58 (m, 1H), 7.45–7.43 (m, 2H), 7.35 (t, *J* = 7.6 Hz, 2H), 7.30–7.28 (m, 3H), 7.23–7.21 (m, 1H), 5.22 (s, 1H), 3.82 (m, *J* = 13.6 Hz, 1H), 3.72–3.55 (m, 5H), 3.19–3.13 (m, 1H), 2.96–2.91 (m, 1H). ^13^C NMR (75 MHz, CDCl_3_): δ 167.5, 138.3, 134.2, 128.7 (4C), 128.4, 127.7, 127.6, 127.1 (2C), 124.2, 78.3, 56.7, 50.6, 41.7, 38.8. HRMS (MALDI-FT ICR): *m*/*z* calcd for [C_18_H_18_N_2_O + H]^+^: 279.1492, found: 279.1496. IR (KBr): 1747, 681, 621 cm^−1^.

***2,3,4,5,8,12b-hexahydroazepino [2,1-a]isoquinolin-7(1H)-one 14f***

Reaction carried out for 72 h (EtOH, 1 mL for 0.28 mmol). Yellow solid. Yield: 78%, 48 mg. M.p. 68 °C (dec.). Rf. 0.35 (DCM 100/MeOH 1, SiO_2_). ^1^H NMR (400 MHz, CDCl_3_): δ 7.43–7.40 (m, 1H), 7.26–7.24 (m, 2H), 7.14–7.12 (m, 1H), 5.25 (s, 1H), 4.47–4.41 (m, 1H), 3.72 (d, *J* = 19.2 Hz, 1H), 3.56 (d, *J* = 19.2 Hz, 1H), 3.08–2.99 (m, 2H), 2.89–2.83 (m, 1H), 1.92 (br s, 1H), 1.88–1.79 (m, 2H), 1.73–1.64 (m, 2H). ^13^C NMR (100 MHz, CDCl_3_): δ 169.3, 134.9, 132.1, 128.3, 127.3, 126.8, 126.3, 74.5, 46.3, 44.2, 37.2, 30.0, 27.3. HRMS (MALDI-FT ICR): *m*/*z* calcd for [C_13_H_16_N_2_O + H]^+^: 217.1335; found: 217.1337. IR (KBr): 3150, 1710, 688, 650 cm^−1^.

***9-nitro-2,3,6,10b-tetrahydroimidazo [2,1-a]isoquinolin-5(1H)-one 14h***

Red solid. Yield: 94%, 61 mg. M.p. 180 °C (dec.). Rf. 0.25 (DCM 100/MeOH 1, SiO_2_). ^1^H NMR (400 MHz, CDCl_3_): δ 7.39 (s, 1H), 8.17 (d, *J* = 8.0 Hz, 1H), 7.38 (d, *J* = 8.4 Hz, 1H), 5.39 (s, 1H), 3.75 (d, *J* = 18.4 Hz, 1H), 3.69–3.61 (m, 2H), 3.54–3.50 (m, 1H), 3.42–3.36 (m, 1H), 3.33–3.28 (m, 1H), 2.10 (br s, 1H). ^13^C NMR (100 MHz, CDCl_3_): δ 164.9, 147.6, 139.7, 136.2, 128.5, 123.8, 119.8, 73.6, 45.2, 44.7, 38.8. HRMS (MALDI-FT ICR): *m*/*z* calcd for [C_11_H_11_N_3_O_3_ + H]^+^: 234.0873; found: 234.0876. IR (KBr): 3260, 1712, 1578, 680, 650 cm^−1^.

***10-fluoro-1-methyl-2,3,6,10b-tetrahydroimidazo [2,1-a]isoquinolin-5(1H)-one 14i***

Yellow solid. Yield: 52 mg, 92%. M.p. 177 °C (dec.). Rf. 0.35 (DCM 100/MeOH 1, SiO_2_). ^1^H NMR (400 MHz, CDCl_3_): δ 7.33–7.28 (m, 1H), 7.02–6.97 (m, 2H), 5.37 (s, 1H), 3.98–3.90 (m, 1H), 3.71 (d, *J* = 20.0 Hz, 1H), 3.69 (d, *J* = 20.0 Hz, 1H), 3.37–3.27 (m, 2H), 3.21–3.15 (m, 1H), 2.07 (s, 3H). ^13^C NMR (100 MHz, CDCl_3_): δ 166.3, 159.5 (d, *J_C-F_ =* 249 Hz), 134.8 (d, *J_C-F_ =* 3.5 Hz), 130.4 (d, *J_C-F_ =* 8.9 Hz), 123.5 (d, *J_C-F_ =* 3.2 Hz), 117.3 (d, *J_C-F_ =* 14.3 Hz), 114.2 (d, *J_C-F_ =* 21.5 Hz), 77.0, 53.1, 40.4, 36.7, 36.2. ^19^F NMR (377 MHz, CDCl_3_): δ −121.2. HRMS (MALDI-FT ICR): *m*/*z* calcd for [C_12_H_13_N_2_FO + H]^+^: 221.1085; found: 221.1091. IR (KBr): 1720, 688, 651 cm^−1^.

***2,3-diphenyl-2,3,6,10b-tetrahydroimidazo [2,1-a]isoquinolin-5(1H)-one 14j***

Reaction carried out for 72 h at 80 °C. White crystals (CHCl_3_/Hexane). Yield: 78%, 74 mg. M.p. 112–113 °C. Rf. 0.67 (DCM 100/MeOH 1, SiO_2_). ^1^H NMR (400 MHz, CDCl_3_): δ 7.74 (app t, *J* = 7.4 Hz 1H), 7.37–7.29 (m, 4H), 7.18–7.14 (m, 6H), 6.88–6.82 (m, 3H), 6.02 (s, 1H), 4.43 (d, *J* = 6.0 Hz 1H), 4.49 (m, 1H), 3.78 (d, *J* = 13.7 Hz, 1H), 3.65 (d, *J* = 16.8 Hz, 1H), 3.20 (br s, 1H). ^13^C NMR (100 MHz, CDCl_3_): δ 168.2, 136.8, 136.7, 135.4, 135.3, 132.9, 128.5, 128.2 (2C), 127.9 (2C), 127.7, 127.5, 127.4, 127.2 (2C), 127.1 (2C), 123.4, 74.2, 64.4, 63.8, 39.8. HRMS (MALDI-FT ICR): *m*/*z* calcd for [C_23_H_20_N_2_O + H]^+^: 341.1648; found: 341.1647. IR (KBr): 1720, 790, 686, 650 cm^−1^.

***1,1′-(ethane-1,2-diyl)bis(2,3,6,10b-tetrahydroimidazo [2,1-a]isoquinolin-5(1H)-one 14k***

To a solution of aldehyde **9** (50 mg, 0.28 mmol, 2 eq.) in EtOH (500 µL), triethylenetetramine (0.14 mmol, 1 eq.) was added, and the mixture was stirred at 50 °C for 24 h. The solvent was evaporated, and the crude was purified on neutral alumina (from Pentane 20% in CH_2_Cl_2_ to CH_2_Cl_2_). Mixture of diasteroisomers (dr 60/40). Yellow solid. Yield: 35 mg, 62%. Rf. 0.35 (DCM 100/MeOH 1, SiO_2_). ^1^H NMR (400 MHz, CDCl_3_): δ 7.59 (s, 2H), 7.36–7.30 (m, 6H), 5.23 (s, 1H), 5.17 (s, 1H), 3.82–3.49 (m, 8H), 3.48–3.42 (m, 2H), 3.08–3.04 (m, 3H), 2.95–2.84 (m, 3H). ^13^CNMR (100 MHz, CDCl_3_): δ 167.2, 167.1, 134.4, 133.9, 132.8, 132.7, 128.4, 128.3, 127.7 (2C), 126.9 (2C), 124.2, 123.9, 78.9, 78.7, 52.9, 52.3, 52.1, 51.7, 41.8, 41.7, 38.8, 38.6. HRMS (MALDI-FT ICR): *m*/*z* calcd for [C_24_H_26_N_4_O_2_ + H]^+^: 403.2128; found: 403.2141. IR (KBr): 1730, 1714, 690, 652 cm^−1^.

### 3.2. General Procedure for the Synthesis of Tetracyclic Lactams ***18***, ***19***, ***20a***–***20e***

To a solution of aldehyde **9** (50 mg, 0.28 mmol) in EtOH (500 µL), diamine **15**, **16**, or **17** (0.28 mmol, 1 eq.) was added, and the mixture was stirred at room temperature for 45–120 min. The solvent was evaporated, and the crude was purified on a short column of silica (Ethyl Acetate 10% in Pentane).

***8,13,14,14a-tetrahydrobenzo [5,6][1,3]diazepino [2,1-a]isoquinolin-6(5H)-one 18***

Reaction carried out for 2 h. Tan solid. Yield: 67 mg, 95%. M.p. 180 °C (dec.). Rf. 0.20 (Pentane 90/Ethyl Acetate 10, SiO_2_).^1^H NMR (400 MHz, CDCl_3_): δ 7.46 (app t, *J* = 7.4 Hz, 2H), 7.28–7.16 (m, 4H), 7.12–7.06 (m, 2H), 5.64 (s, 1H), 5.40 (d, *J* = 14.4 Hz, 1H), 4.43 (d, *J* = 15.6 Hz, 1H), 3.31 (d, *J* = 14.8 Hz, 1H), 4.08 (d, *J* = 16.0 Hz, 1H), 3.60 (d, *J* = 21.6 Hz, 1H), 3.55 (d, *J* = 22.4 Hz, 1H). ^13^C NMR (100 MHz, CDCl_3_): δ 167.9, 140. 9, 137.7, 133.2, 131.4, 130.0, 128.4, 128.3, 127.6, 127.5, 127.4, 127.3, 126.9, 78.9, 52.9, 50.5, 35.7. HRMS (MALDI-FT ICR): *m*/*z* calcd for [C_17_H_16_N_2_O + H]^+^: 265.1335; found: 265.1331. IR (KBr): 3319, 1710, 688, 650 cm^−1^

***benzo [4,5]imidazo [2,1-a]isoquinolin-6(5H)-one 19***

Reaction carried out for 24 h. Yellow solid. Yield: (23 mg, 35%). M.p. 147–150 °C. Rf. 0.50 (Pentane 90/Ethyl Acetate 10, SiO_2_). Spectra are in agreement with literature data [18]. ^1^H NMR (400 MHz, CDCl_3_) *δ* 8.44–8.37 (m, 1H), 8.29 (d, *J* = 6.7 Hz, 1H), 7.79 (d, *J* = 6.6 Hz, 1H), 7.52–7.36 (m, 4H), 7.35–7.27 (m, 1H), 4.19 (s, 2H). HRMS (MALDI-FT ICR: *m*/*z* calcd for [C1_5_H_11_N_2_O + H]^+^: 235.0866; found: 235.0864.

***5,8,13,13a- tetrahydro -6H-isoquinolino [1,2-b]quinazolin-6-one 20a***

Reaction carried out for 45 min. White solid. Yield: 67 mg, 95%. M.p. 218°C (dec.). Rf. 0.40 (Pentane 90/Ethyl Acetate 10, SiO_2_) ^1^H NMR (400 MHz, CDCl_3_): δ 7.42–7.33 (m, 3H), 7.20 (d, *J* = 7.2 Hz, 1H), 7.08 (t, *J* = 7.2 Hz, 2H), 6.87 (t, *J* = 7.6 Hz, 1H), 6.67 (d, *J* = 8.0 Hz, 1H), 5.79 (s, 1H), 5.65 (d, *J* = 20.8 Hz, 1H), 4.36 (d, *J* = 16.8 Hz, 1H), 4.07 (br s, 1H, NH), 3.80 (d, *J* = 20.8 Hz, 1H), (3.70 (d, *J* = 20.8 Hz, 1H). ^13^C NMR (100 MHz, CDCl_3_): δ 167.5, 142.7, 131.8, 130.2, 129.1, 127.8, 127.4, 127.1, 127.0, 126.3, 120.5, 120.4, 116.9, 68.7, 43.9, 35.2. HRMS (MALDI-FT ICR): *m*/*z* calcd for [C_16_H_15_N_2_O + H]^+^: 251.1178; found: 251.1188. IR (KBr): 3310, 1715, 689, 676 cm^−1^. The reaction was scaled up to 1.5 mmols, achieving 92% yield.

***2-nitro-5,8,13,13a-tetrahydro-6H-isoquinolino [1,2-b]quinazolin-6-one 20b***

Reaction carried out for 1 h. Red solid. Yield: 76 mg, 92%. M.p. 178 °C (dec.). Rf. 0.2 (Pentane 90/Ethyl Acetate 10, SiO_2_) ^1^H NMR (400 MHz, DMSO-*d*_6_): δ 8.37 (s, 1H), 8.20 (d, *J* = 8.4 Hz, 1H), 7.50 (d, *J* = 8.8 Hz, 1H), 7.01 (d, *J* = 7.6 Hz, 1H), 6.96 (t, *J* = 7.6 Hz, 1H), 6.68 (t, *J* = 7.6 Hz, 1H), 6.62 (*J* = 8.0 Hz, 1H), 6.36 (s, 1H), 5.97 (s, 1H), 5.31 (d, *J* = 16.4 Hz, 1H), 4.28 (*J* = 16.4 Hz, 1H), 3.84 (d, *J* = 23.2 Hz, 1H), 3.77 (*J* = 23.62 Hz, 1H). ^13^C NMR (150 MHz, DMSO-*d*_6_): δ 168.7, 147.9, 145.6, 142.4, 134.3, 131.0, 128.9, 128.5, 125.3, 124.3, 121.5. 120.7, 120.0, 118.2, 69.0, 45.3, 37.1. HRMS (MALDI-FT ICR): *m*/*z* calcd for [C_16_H_13_N_3_O_3_ + H]^+^: 296.1030; found: 296.1037. IR (KBr): 3240, 1710, 1580, 680, 670 cm^−1^.

***2-bromo-5,8,13,13a-tetrahydro-6H-isoquinolino [1,2-b]quinazolin-6-one 20c***

Reaction carried out for 1 h. White solid. Yield: 81 mg, 89%. M.p. 230 °C (dec.). Rf. 0.55 (Pentane 90/Ethyl Acetate 10, SiO_2_)^1^H NMR (400 MHz, CDCl_3_): δ 7.58 (s, 1H), 7.50 (d, *J* = 8.0 Hz, 1H), 7.09 (d, *J* = 7.6 Hz, 3H), 6.89 (t, *J* = 7.6 Hz, 1H), 6.69 (d, *J* = 8.0 Hz, 1H), 5.75 (s, 1H), 5.63 (d, *J* = 16.8 Hz, 1H), 4.35 (*J* = 16.8 Hz, 1H), 4.01 (br s, 1H), 3.74 (d, *J* = 20.8 Hz, 1H), 3.66 (*J* = 20.8 Hz, 1H). ^13^C NMR (150 MHz, CDCl_3_): δ 168.4, 143.5, 133.8, 133.7, 132.4, 131.0, 131.8, 128.9, 129.6, 122.3, 122.1, 122.0, 118.8, 69.7, 45.4, 36.3. HRMS (MALDI-FT ICR): *m*/*z* calcd for [C_16_H_13_BrN_2_O + H]^+^: 329.0284; found: 329.0282. IR (KBr): 3230, 1700, 687 cm^−1^.

***1-fluoro-5,8,13,13a-tetrahydro-6H-isoquinolino [1,2-b]quinazolin-6-one 20d***

Reaction carried out for 1 h. White solid. Yield: 68 mg, 91%. M.p. 218 °C (dec.). Rf. 0.45 (Pentane 90/Ethyl Acetate 10, SiO_2_) ^1^H NMR (600 MHz, CDCl_3_): δ 7.32–7.29 (m, 1H), 7.04–6.95 (m, 4H), 6.81 (dt, *J*_1_ = 7.8 Hz, *J*_2_ = 1.2 Hz, 1H), 6.60 (dd, *J*_1_ = 7.8 Hz, *J*_2_ = 0.6 Hz, 1H), 6.00 (s, 1H), 5.62 (d, *J* = 16.2 Hz, 1H), 4.33 (d, *J* = 16.2 Hz, 1H), 4.16 (br s, 1H), 3.76 (d, *J* = 21.1 Hz, 1H), 3.76 (d, *J* = 20.4 Hz, 1H), ^13^C NMR (150 MHz, CDCl_3_): δ 168.7, 160.9 (d, *J_C-F_* = 246 Hz), 144.3, 136.3 (d, *J_C-F_* = 2.7 Hz), 132.2, (d, *J_C-F_* = 8.55 Hz), 128.9 (*J_C-F_* = 54.0 Hz), 124.9 (*J_C-F_* = 3.0 Hz), 121.8, 121.3, 117.8 (2C), 119.7 (d, *J_C-F_* = 15.3 Hz), 115.0 (d, *J_C-F_* = 93 Hz), 65.4 (d, *J_C-F_* = 3.75 Hz), 45.4, 36.5. ^19^F NMR (377 MHz, CDCl_3_): δ −116.1. HRMS (MALDI-FT ICR): *m*/*z* calcd for [C_16_H_13_FN_2_O + H]^+^: 269.1085; found: 269.1055. IR (KBr): 3210, 1710, 678 cm^−1^.

***3-chloro-5,8,13,13a-tetrahydro-6H-isoquinolino [1,2-b]quinazolin-6-one 20e***

Reaction carried out for 1.5 h. White solid. Yield: 71 mg, 90%. M.p. 198 °C (dec.). Rf. 0.50 (Pentane 90/Ethyl Acetate 10, SiO_2_) ^1^H NMR (600 MHz, CDCl_3_): δ 7.40 (d, *J* = 7.8 Hz, 1H), 7.33 (d, *J* = 8.4 Hz, 1H), 7.23 (s, 1H), 7.11 (t, *J* = 6.0 Hz, 2H), 6.92 (d, *J* = 7.2 Hz, 1H), 6.70 (d, *J* = 8.4 Hz, 1H), 5.79 (s, 1H), 5.65 (d, *J* = 16.8 Hz, 1H), 4.38 (*J* = 16.8 Hz, 1H), 3.82 (d, *J* = 20.4 Hz, 1H), 3.66 (*J* = 21.0 Hz, 1H). ^13^C NMR (150 MHz, CDCl_3_): δ 168.3, 136.6, 135.4, 130.1, 129.3, 129.2, 128.95, 128.9 (2C), 128.6, 122.5, 122.5, 118.8, 69.8, 45.4, 36.3. HRMS (MALDI-FT ICR): *m*/*z* calcd for [C_16_H_13_ClN_2_O + H]^+^: 285.0789; found: 285.0756. IR (KBr): 3236, 1708, 684 cm^−1^.

### 3.3. Follow-Up Chemistry

#### 3.3.1. Reduction Using BH_3_(CH_3_)_2_S

***2-((3,4-dihydroisoquinolin-2(1H)-yl)methyl)aniline 21***

Under a nitrogen atmosphere, to a suspension of **20a** (60 mg, 0.24 mmol, 1 equiv.) in THF (3.0 mL), BH_3_(CH_3_)_2_S (10 M, 240 µL, 10 equiv.) was added dropwise. The reaction mixture was refluxed for 1 h. The reaction mixture was cooled to room temperature, and then the solvent and dimethyl sulfide were evaporated. After the addition of 6 N HCl (1.5 mL) to the residue, the solution was heated at 80 °C for 1 h and then cooled to room temperature. The mixture was neutralized with 4 N NaOH and extracted with CH_2_Cl_2_. The organic layer was washed with brine and dried over MgSO_4_. After concentration, the product **19** was isolated by chromatography on silica (from Ethyl Acetate 30% in Pentane to 50%). Pale oil. Yield: 54 mg, 94%. Rf. 0.45 (Pentane 70/Ethyl Acetate 30, SiO_2_). ^1^H NMR (400 MHz, CDCl_3_): δ 7.12–7.10 (m, 4H), 7.05 (d, *J* = 7.6 Hz, 1H), 6.99 (d, *J* = 7.2 Hz, 1H), 6.70 (t, *J* = 7.2 Hz, 1H), 6.64 (d, *J* = 7.8 Hz, 1H), 3.68 (s, 2H), 3.60 (s, 2H), 2.88 (t, *J* = 5.70 Hz, 2H), 2.73 (t, *J* = 5.70 Hz, 2H). ^13^C NMR (100 MHz, CDCl_3_): δ 147.2, 134.9, 134.3, 130.5, 128.7, 128.5, 126.7, 126.2, 125.7, 122.3, 117.6, 115.6, 62.0, 55.8, 50.3, 29.4. HRMS (MALDI-FT ICR): *m*/*z* calcd for [C_16_H_18_N_2_ + H]^+^: 239.1543; found: 239.1504. IR (neat): 3236, 789, 684, 678 cm^−1^.

#### 3.3.2. Reduction Using LiAlH_4_

***5,8,13,13a-tetrahydro-6H-isoquinolino [1,2-b]quinazoline 22* [30]**

To a suspension of **20a** (60 mg, 0.24 mmol, 1 equiv.) in THF (4 mL) in an ice bath at 0 °C, a solution of LiAlH4 1 M (480 µL, 2 equiv.) in THF was added, and the reaction was stirred at the same temperature for 1 h. Then, the solvent was evaporated, and the mixture was neutralized with 4 N NaOH and extracted with CH_2_Cl_2_. The crude material was purified by chromatography on silica gel (diethyl ether 50% in petroleum ether) to give **22** as a viscous colorless oil. Yield: 89%, 50 mg. Spectra are in agreement with the literature data [30,31]. Rf. 0.35 (Et_2_O 50/petroleum ether 50, SiO_2_). ^1^H NMR (400 MHz, CDCl_3_): δ 7.35 (d, *J* = 7.2 Hz, 1H), 7.30–7.23 (m, 2H), 7.18 (dd, *J* = 6.8 Hz, 1H 1H), 7.05 (t, *J* = 8.0 Hz, 1H), 6.99 (d, *J* = 7.2 Hz, 1H), 6.74 (t, *J* = 7.2 Hz, 1H), 6.57 (d, *J* = 8.4 Hz, 1H), 5.15 (s, 1H), 4.34 (d, *J* = 16.0 Hz, 1H), 3.86 (d, *J* = 16.0 Hz, 1H), 3.86 (br s, 1H), 3.22–3.17 (m, 1H), 3.04–2.94 (m, 1H), 2.74–2.68 (m, 1H). HRMS (MALDI-FT ICR): *m*/*z* calcd for [C_16_H_18_N_2_ + H]^+^: 237.1386; found: 237.1383.

## 4. Conclusions

In summary, we developed an effective one-pot approach for the synthesis of novel polycyclic fused δ-lactams from readily available aldehydes and diamines under catalyst-free and green conditions. This procedure, which combines a sequential aza-Mannich/lactamization cascade, was easily scalable, affording a variety of compounds analogous to natural products and pharmaceuticals, further emphasizing the versatility of the developed methodology. Follow-up chemistry will also be explored to enable access to other bioactive molecules. Further studies are in course to expand the scope of this method using amino alcohols and aminothiols, which may lead to new classes of polyheterocyclic compounds.

## Figures and Tables

**Table 1:** Reaction conditions investigation of the aza-Mannich/lactamization process.

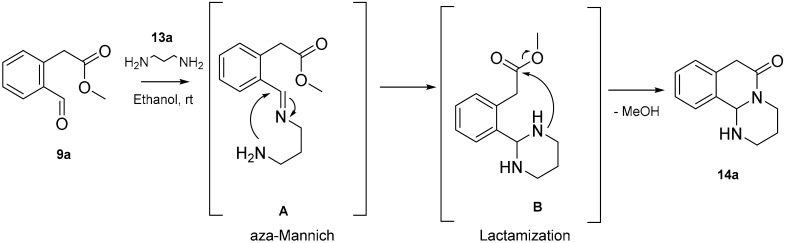			
**Entry**	**[M] 9a**	**Time**	**Yield % ^a^**
1	0.56	2 h	50% ^b^
2	0.56	2 h	52% ^c^
3	0.56	2 h	73% ^d^
4	0.28	2 h	74% ^d^
5	0.56	24 h	** 95 ** % ^d^
6	0.28	24 h	88% ^d^
7	0.14	24 h	57% ^d^
8	0.56	2 h	71% ^d,e^

^a^ Isolated yield by chromatography. ^b^ Purification by silica. ^c^ Purification by silica/Et_3_N 1%. ^d^ Purification by neutral alumina. ^e^ Reaction performed at 60 °C. The best result is highlighted in bold.

## Data Availability

CCDC 2452206 Contains the Supplementary Crystallographic Data for Compound **14j** of This Paper. These Data Can Be Obtained Free of Charge from The Cambridge Crystallographic Data Centre. See Supporting Information for Further Details. Available online: http://www.ccdc.cam.ac.uk/structures (accessed on 18 June 2025).

## Supplementary Materials

The following supporting information can be downloaded at: https://www.mdpi.com/article/10.3390/molecules30132702/s1. Refs. [23,33,34,35,36,37,38,39,40] are cited in the Supplementary Materials.
