# Supplementary material for: Catalyst-Free Spontaneous Aza-Mannich/Lactamization Cascade Reaction: Easy Access to Polycyclic δ-Lactams
Source: Molecules. 2025 Jun 23;30(13):2702. doi: 10.3390/molecules30132702 (PMC12251525; doi:10.3390/molecules30132702)

Supporting Information

Catalyst-free spontaneous aza-Mannich/lactamization cascade reaction: easy access to polycyclic δ-lactams

Antonia Di Mola,* Caterina Vietri, Consiglia Tedesco and Antonio Massa*

Dipartimento di Chimica e Biologia "A. Zambelli", Università degli Studi di Salerno, Via Giovanni Paolo II, 84084 Fisciano, Italy

* Correspondence: adimola@unisa.it; amassa@unisa.it

Table of Contents

1. General remarks 2
2. Synthesis of starting materials 2
3. General procedure for the synthesis of polycyclic lactams 3
4. X-ray Crystallography 4
5. References 6
6. Copies of NMR Spectra 7

1. General

Unless otherwise noted, all chemicals, reagents and solvents for the performed reactions are commercially available. Aldehydes **9** were prepared according to the literature procedures.[1] All the reactions were monitored by thin layer chromatography (TLC) on precoated silica gel plates (0.25 mm) and visualized by fluorescence quenching at 254 nm. Flash chromatography was carried out using neutral activated alumina. (Merck, Darmstadt, Germany) or silica. Yields are given for isolated products showing one spot on a TLC plate. The NMR spectra were recorded on Bruker DRX 600, 400, 300 and 250 MHz spectrometers (600 MHz, ^{1}H, 150 MHz, ^{13}C; 400 MHz, ^{1}H, 100.6 MHz; ^{13}C, 300 MHz, ^{1}H, 75.5 MHz, ^{13}C, 250 MHz, ^{1}H, 62.5 MHz, ^{13}C). Internal reference was set to the residual solvent signals (δ_H 7.26 ppm, δ_C 77.16 ppm for $CDCl_3$, δ_H 2.50 ppm, δ_C 39.10 ppm for DMSO-d_6). The ^{13}C NMR spectra were recorded under broad-band proton decoupling. ^{1}HNMR data and HRMS are reported for all compounds. IR and ^{13}CNMR data are given only for unknown compounds. The following abbreviations are used to indicate the multiplicity in NMR spectra: s singlet, d-doublet, t-triplet, q-quartet, dd-doublet of doublets, m-multiplet, br s-broad signal. High resolution mass spectra (HRMS) were acquired using a Bruker SolariX XR Fourier transform ion cyclotron resonance mass spectrometer (Bruker Daltonik GmbH, Bremen, Germany) equipped with a 7T refrigerated actively shielded superconducting magnet. For ionization of the samples electrospray ionization (ESI) or MALDI was applied. IR spectra were recorded on an IR Bruker Vertex 70v spectrometer.

Synthesis of 4- substituted aldehydes

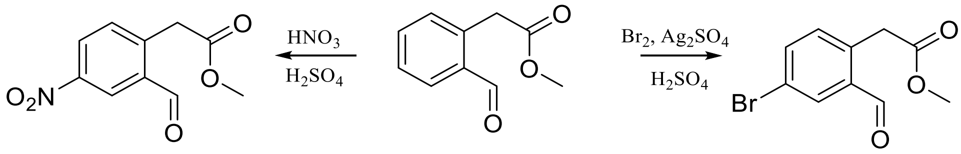

9b **9a** **9c**

Aldehydes were prepared according to the literature procedures.[1]

Synthesis of 5-sustitued aldehydes and 3-substituted aldehydes

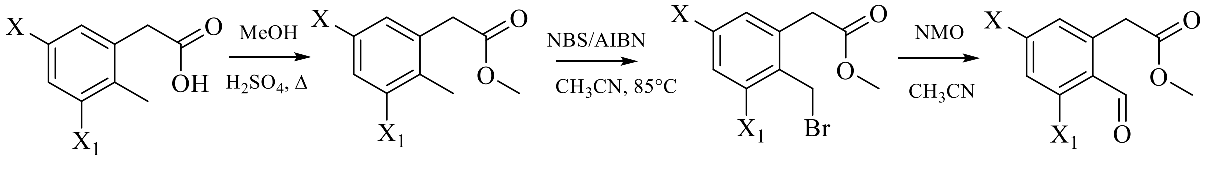

9d, X= H, X_1= F
9e, X= Cl, X_1= H

Aldehydes were prepared according to the literature procedures.[1]

Methyl 2-(3-fluoro-2-formylphenyl)acetate 9d. White solid (91%). M.p. 151-152 °C. ^{1}H NMR (400 MHz, $CDCl_3$): δ 10.48 (s, 1H), 7.55-7.49 (m, 1H), 7.14 (t app, *J*= 9.65 Hz, 1H), 7.04 (d, *J*= 7.6 Hz, 1H), 4.00 (s, 2H), 3.70 (s, 3H). ^{13}C NMR (100 MHz, $CDCl_3$) δ 188.7 (d, J_{C-F}= 10.9 Hz), 171.1, 166.5 (d, J_{C-F}= 257 Hz), 137.2, 135.4 (J_{C-F}= 10.3 Hz), 128.2 (d, J_{C-F}= 3.2 Hz), 122.5 (d, J_{C-F}= 6.1 Hz), 115.7 (d, J_{C-F}= 21.5 Hz), 52.0, 39.3. ^{19}FNMR (377 MHz, $CDCl_3$): δ -121.2. HRMS (MALDI-FT ICR): *m/z* calcd. for $[C_{10}H_9FO_3 + H]^+$: 197.008, found: 197.001.

Methyl 2-(5-chloro-2-formylphenyl)acetate 9e. White solid (93%). M.p. 134-135 °C. ^{1}H NMR (250 MHz, $CDCl_3$): δ 10.06 (s, 1H), 7.77 (d, *J*= 8.25 Hz, 1H), 7.47 (dd, *J*= 8.25, 1.75 Hz, 1H), 7.30 (s, 1H), 4.02 (s, 2H), 3.71 (s, 3H). ^{13}C NMR (62.5 MHz, $CDCl_3$): δ 191.2, 170.4, 139.7, 137.8, 134.9, 132.1, 127.8, 51.8, 30.1. HRMS (MALDI-FT ICR): *m/z* calcd. for $[C_{10}H_9ClO_3 + H]^+$: 213.0313, found: 213.0316.

General Procedure for the synthesis of monobenzylic substituted diamines

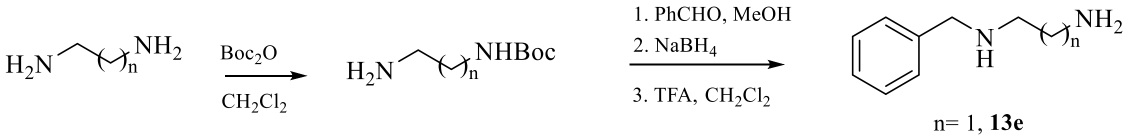

n= 1, **13e**
n= 2, **13b**

N-Benzyl diamines were prepared according to the literature procedures.[2]

Synthesis of *o*-xylilendiamine

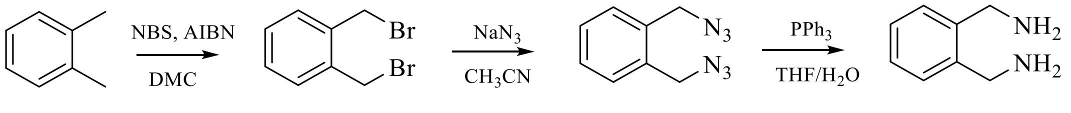

15

O-xylilendiamine was prepared according to the literature procedures.[3]

General procedure for the synthesis of tricyclic lactams 14a-14j

To a solution aldehyde **9** (50 mg, 0.28 mmol) in EtOH (500 μL), diamine **13** (0.28 mmol, 1 eq.) was added and the mixture was stirred at room temperature for 24h. Sol-vent was evaporated and the crude was purified on a short colunm of neutral alumina (from Pentane 20% in CH_2Cl_2 to CH_2Cl_2).

General procedure for the synthesis of dimeric lactams 14k

To a solution aldehyde **9** (50 mg, 0.28 mmol, 2 eq.) in EtOH (500 μL), triethylene-tetramine (0.14 mmol, 1 eq.) was added and the mixture was stirred at 50 °C for 24h. Solvent was evaporated and the crude was purified on neutral alumina (from Pentane 20% in CH_2Cl_2 to CH_2Cl_2).

General procedure for the synthesis of tetracyclic lactams 18, 19, 20a-20e

To a solution aldehyde **9** (50 mg, 0.28 mmol) in EtOH (500 μL), diamine **15** or **16** or **17** (0.28 mmol, 1 eq.) was added and the mixture was stirred at room temperature for 45-120 min. Solvent was evaporated and the crude was purified on a short column of silica (Ethyl Acetate 10% in Pentane).

X-ray crystallography

Crystals of the compound **14j** suitable for single crystal X-ray diffraction analysis were obtained dissolving 5 mg of the compounds in $CHCl_3$/Hexane (0.5 mL, 1/2 ratio).

Several crystals of **14j** were selected and mounted in a cryoloop with paratone oil and data collected under a cold nitrogen flow (Oxford Cryostream 800) at 100 K by means of a Bruker D8 QUEST diffractometer equipped with a PHOTON detector using Cu-Ka radiation (λ= 1.54178 Å).

Several crystals were selected and tested and in the best case the collected diffraction images showed reflections only up to 1.05 Å resolution.

Data indexing was performed using APEX3 software.[4] Data integration and reduction were performed using SAINT.[5] Absorption correction was performed by multi-scan method in SADABS.[6] The structure was solved using SHELXS[7] and refined by means of full matrix least-squares based on F^2 using the program SHELXL.[8] OLEX2 was used as GUI.[9]

There are two crystallographically independent molecules. Moreover, one aromatic ring in the molecule is affected by disorder with three atoms in two possible locations (at least). Thus, non-hydrogen atoms were refined anisotropically with the exception of disordered ones, which were refined isotropically. Hydrogen atoms were positioned geometrically and included in structure factors calculations with the exception of those involved in the hydrogen bond with the carbonylic oxygen atoms C11A and C11B and covalently bound to N2A and N2B atoms, whose coordinates were refined.

ORTEP diagram was drawn using OLEX2 (Figure S1) [x6]. Relevant crystallographic data are reported in Table 1.

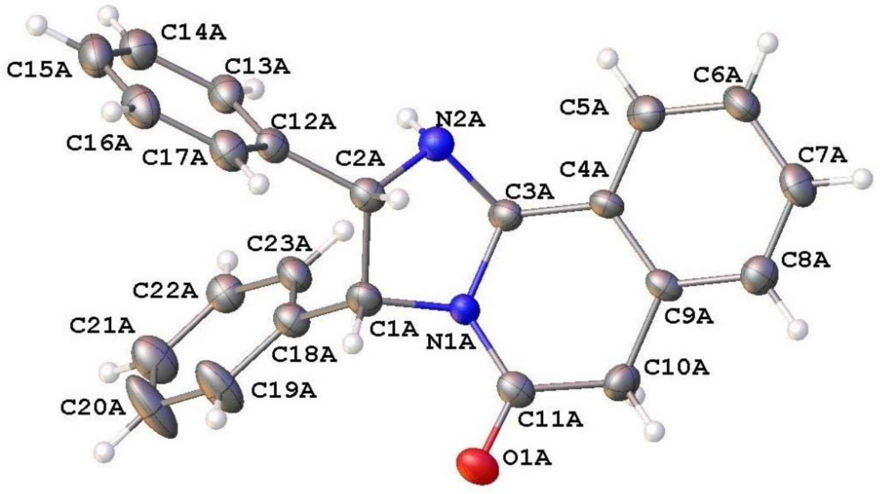

(a)

Figure S1. ORTEP drawing (a) of compound **14j**. Ellipsoids are shown at 50% probability level.

Table S1. Relevant crystallographic data for compound **14j**.

Compound	**14j**
CCDC code	2452206
Empirical formula	$C_{23}H_{20}N_2O$
Formula weight	340.41
Temperature/K	100
Crystal system	triclinic
Space group	P-1
a/Å	6.4768(2)
b/Å	11.9661(4)
c/Å	22.6930(8)
α/°	86.755(2)
β/°	89.778(2)
γ/°	87.749(2)
Volume/Å^3	1754.58(10)

Z	4
ρ_{calc}g/cm^3	1.289
μ/mm^{-1}	0.623
F(000)	720.0
2Θ range for data collection/°	3.9 to 94.474
Index ranges	$-6 \leq h \leq 6$, $-11 \leq k \leq 11$, $-21 \leq l \leq 21$
Reflections collected	15101
Independent reflections	3137 [R_{int} = 0.0502, R_{sigma} = 0.0481]
Data/parameters	3137/474
Goodness-of-fit on F^2	1.125
Final R indexes [I>=2σ (I)]	R_1 = 0.0510, wR_2 = 0.1209
Final R indexes [all data]	R_1 = 0.0789, wR_2 = 0.1453
Largest diff. peak/hole / e Å^{-3}	0.57/-0.33

References

1. Serusi, L.; Di Mola A.; Massa, A. *RSC Adv.* **2023**, *13*, 6557-6563.
2. N'Ta Ambeu, C.; Le Guével , R.; Corlu, A.; Akhanovna Mamyrbekova, J.; Bazureau, J. P. *Molecular Diversity*, **2018**, *22*, 685-708.
3. Gigant, N.; Claveau, E.; Bouyssou, P.; Gillaizeau, I. *Org. Lett.* **2012**, *14*, 844-847.
4. APEX3, version 2015.5-2; Bruker AXS Inc., 2016, Madison, Wisconsin, USA.
5. SAINT, version 8.34A; Bruker AXS Inc., 2013, Madison, Wisconsin, USA.
6. SADABS, version 2014/5; Bruker AXS Inc., 2014, Madison, Wisconsin, USA.
7. Sheldrick, G.M. A short history of SHELX. *Acta Cryst.* **2008**, *A64*, 112–122.
8. Sheldrick, G.M. Crystal structure refinement with SHELXL. *Acta Cryst.* **2015**, *C71*, 3–8.
9. Dolomanov, O.V.; Bourhis, L.J.; Gildea, R.J.; Howard, J.A.K.; Puschmann, H. OLEX2: a complete structure solution, refinement and analysis program *J. Appl. Cryst.*, **2009**, *42*, 339-341.

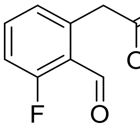

9d

^{1}H NMR (400 MHz, $CDCl_3$)

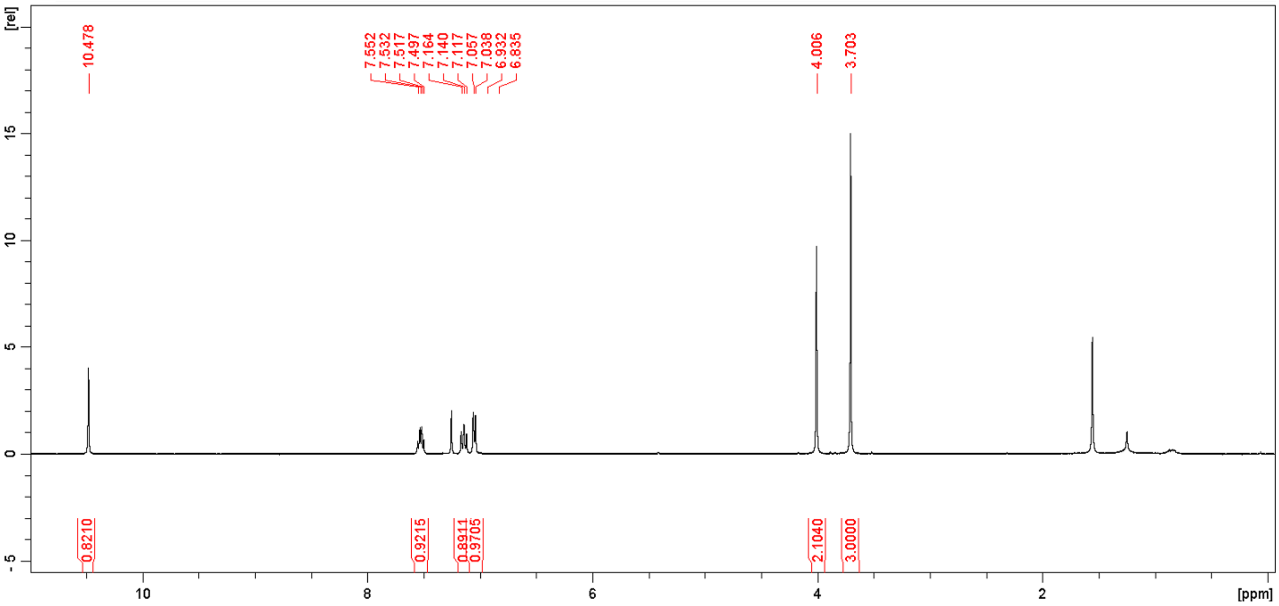

^{13}C{^{1}H} NMR (100 MHz, $CDCl_3$)

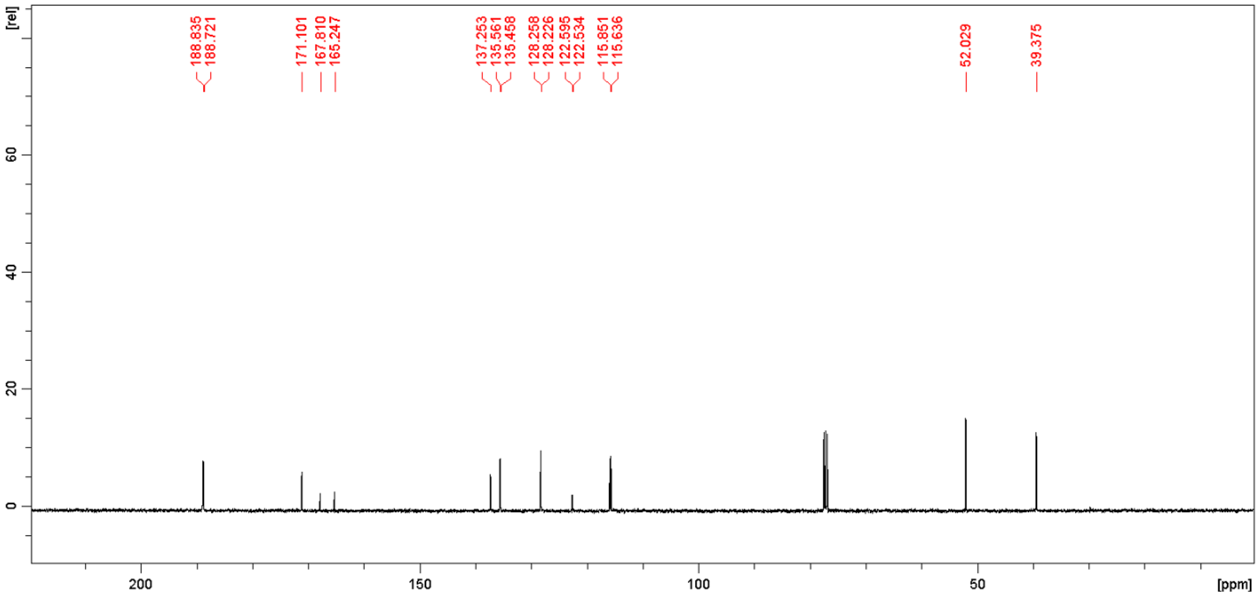

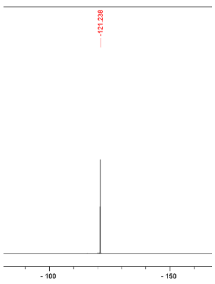

^{19}F NMR

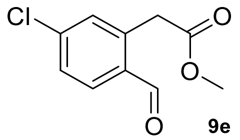

9e

^{1}H NMR (250 MHz, $CDCl_3$)

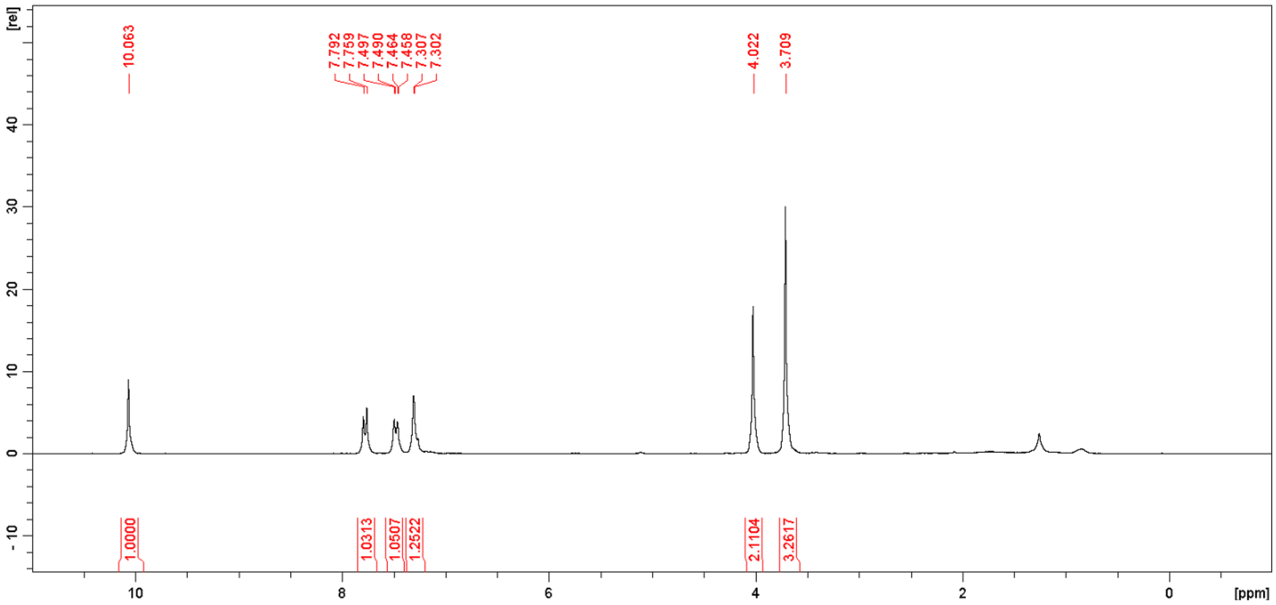

^{13}C{^{1}H} NMR (62.5 MHz, $CDCl_3$)

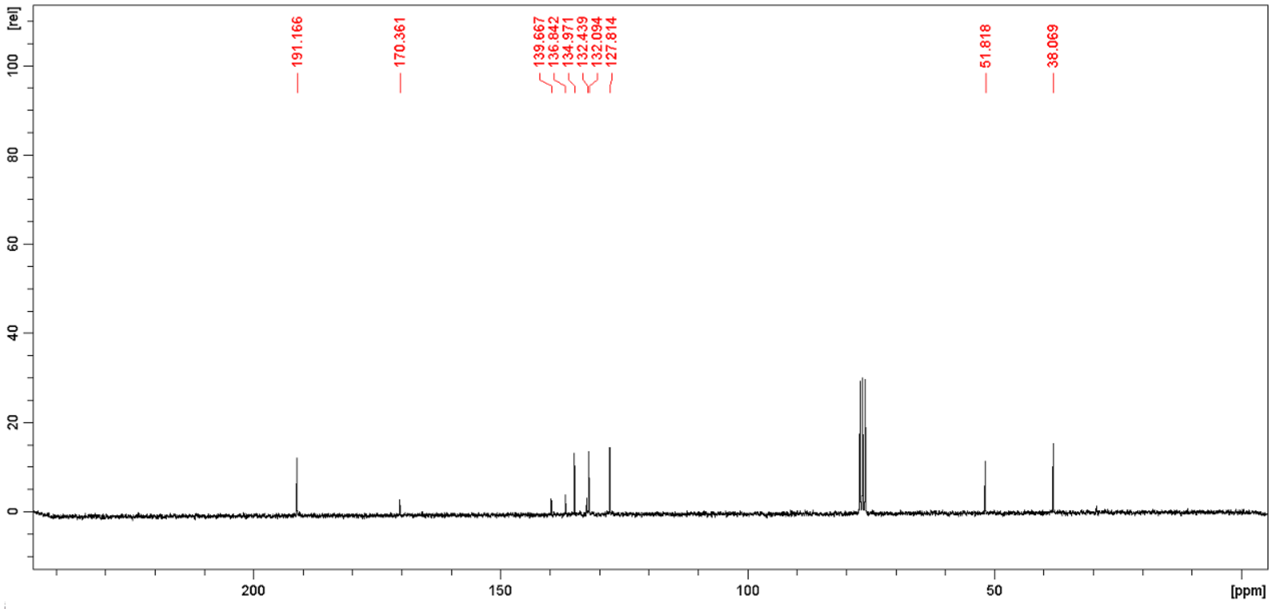

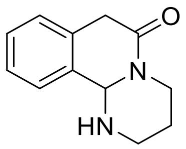

14a

^{1}H NMR (400 MHz, $CDCl_3$)

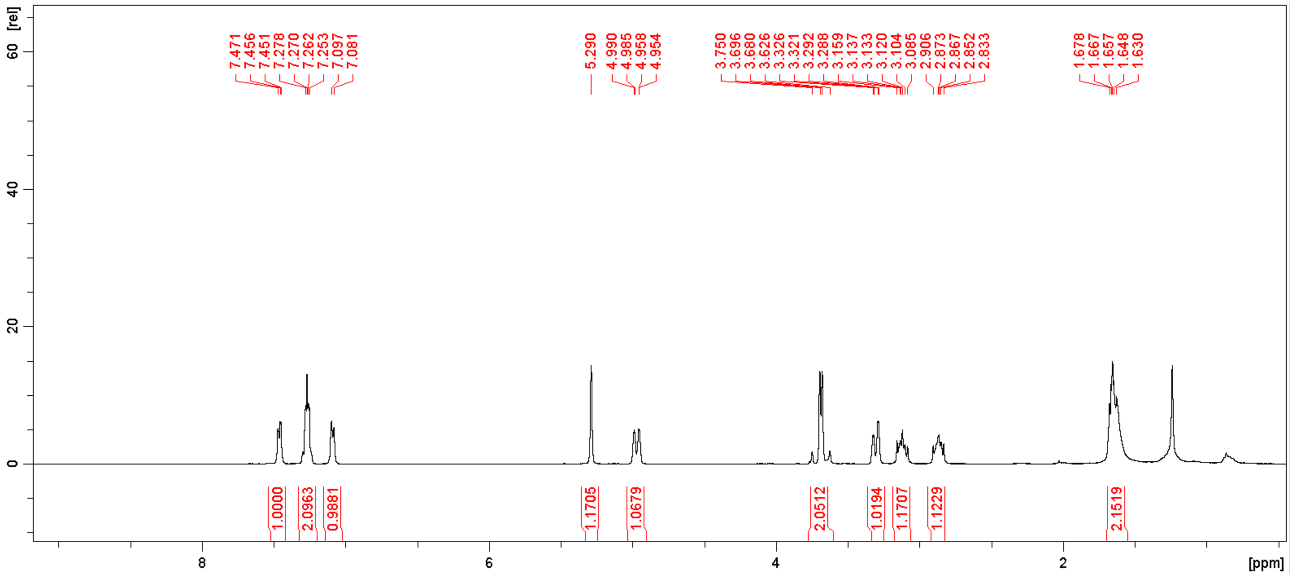

$^{13}C\{^{1}H\}$ NMR (100 MHz, $CDCl_3$)

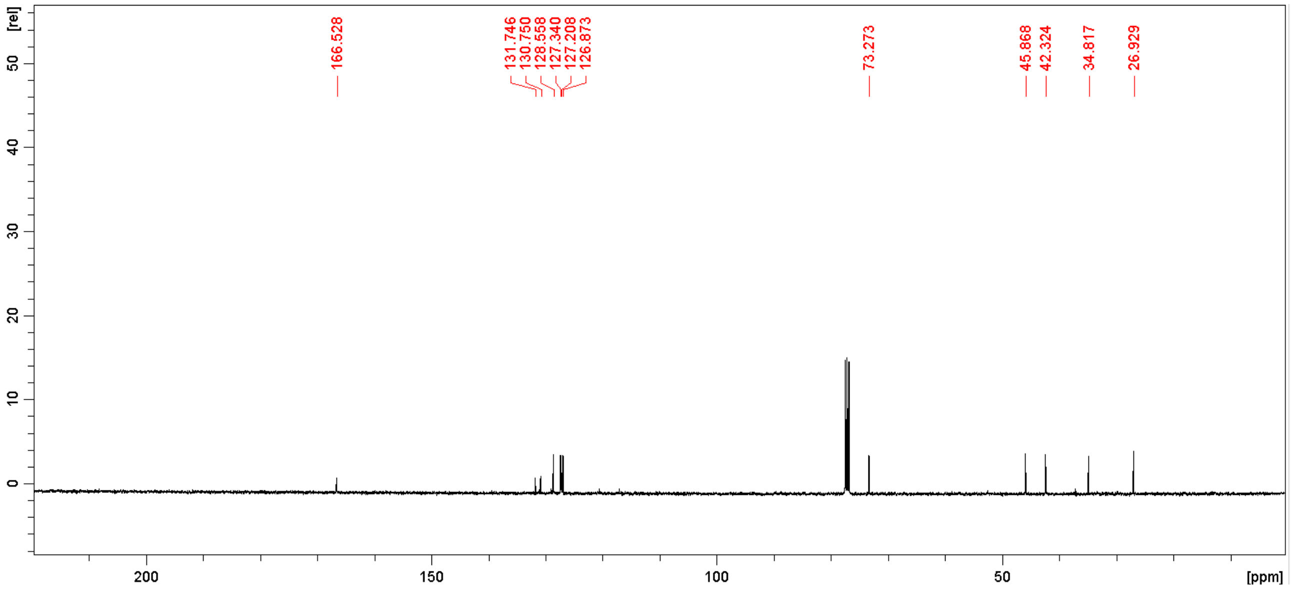

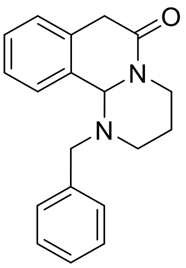

14b

^{1}H NMR (400 MHz, $CDCl_3$)

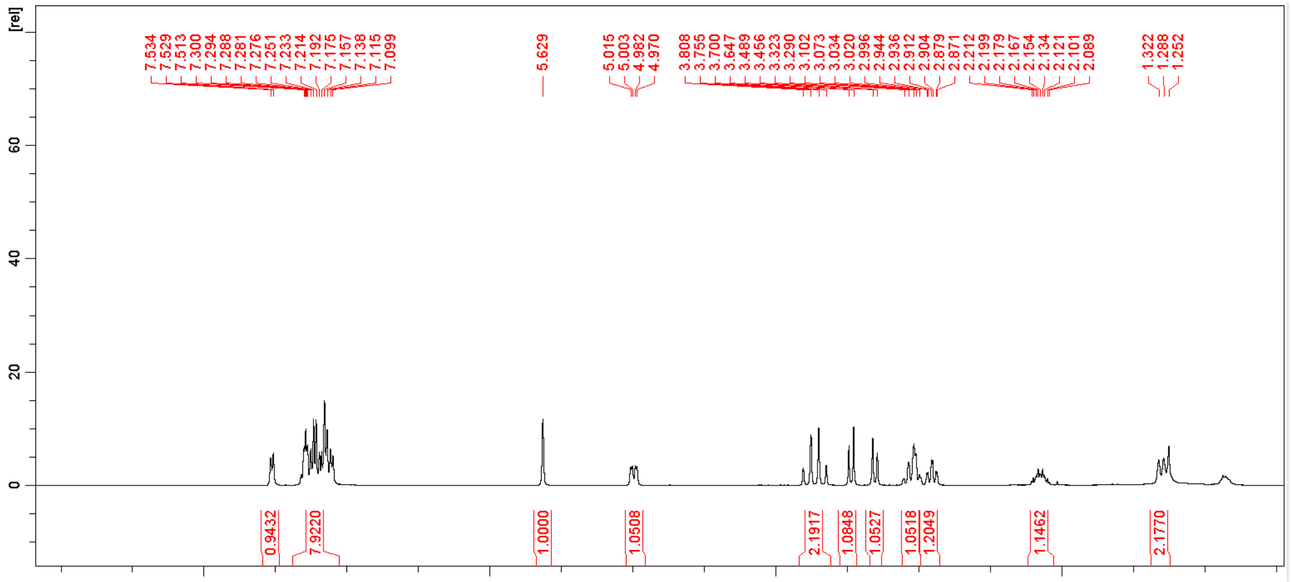

^{13}C{^{1}H} NMR (100 MHz, $CDCl_3$)

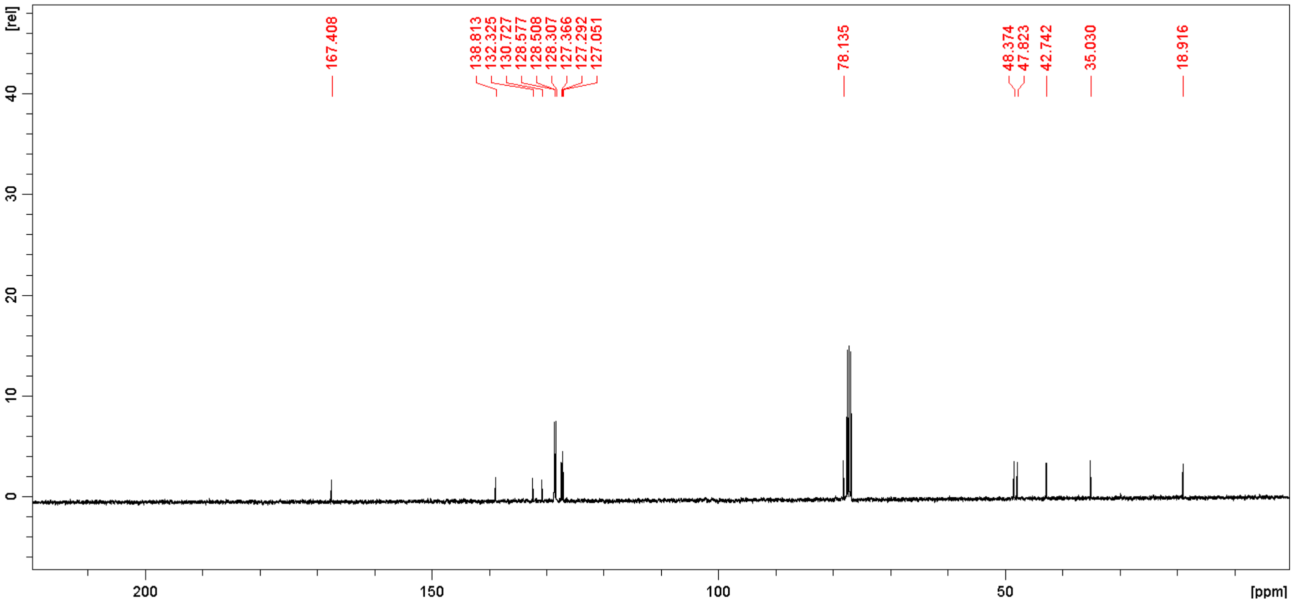

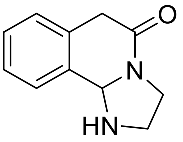

14c

^{1}H NMR (400 MHz, $CDCl_3$)

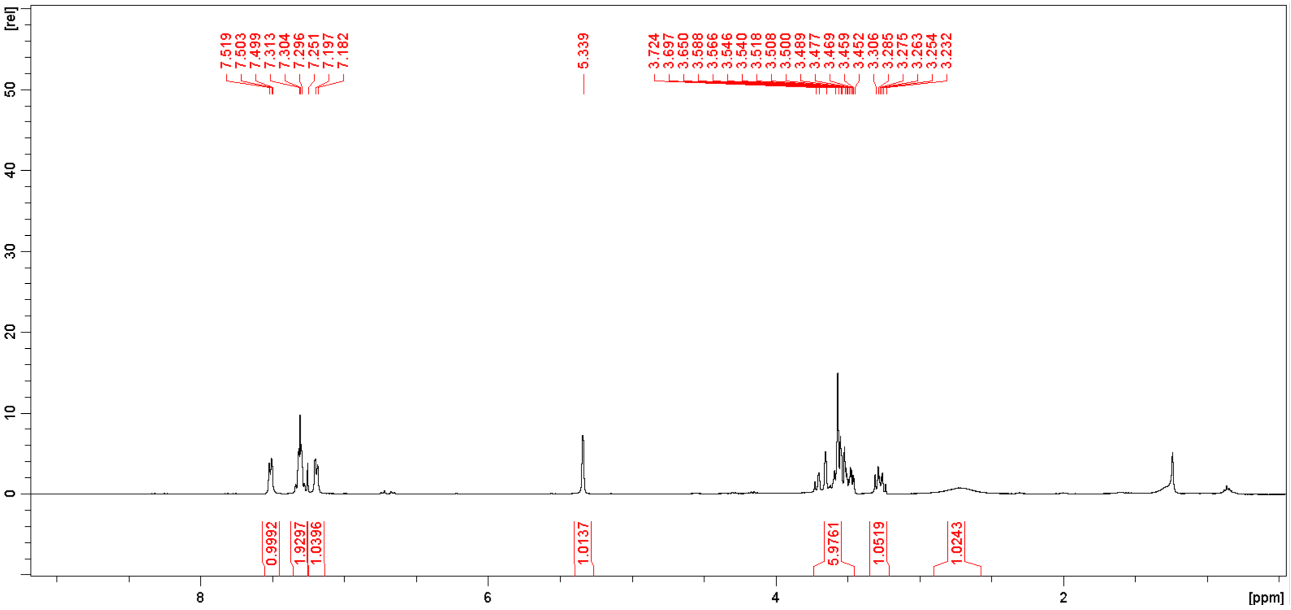

$^{13}C\{^1H\}$ NMR (75 MHz, $CDCl_3$)

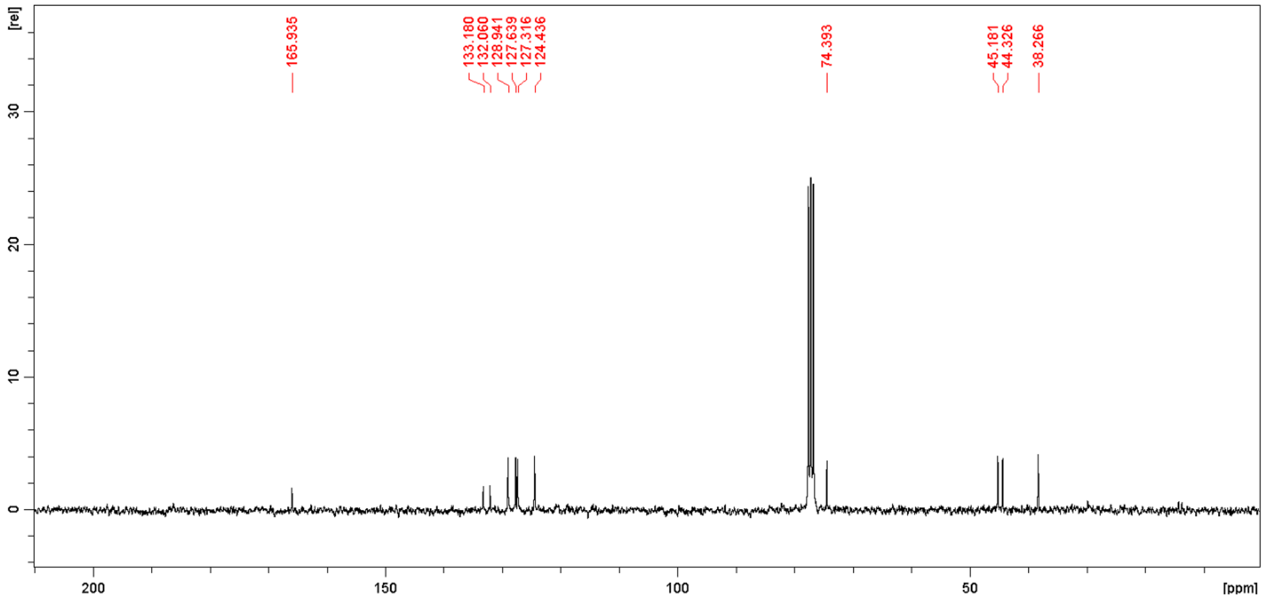

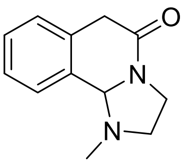

14d

^{1}H NMR (400 MHz, CDCl$_3$)

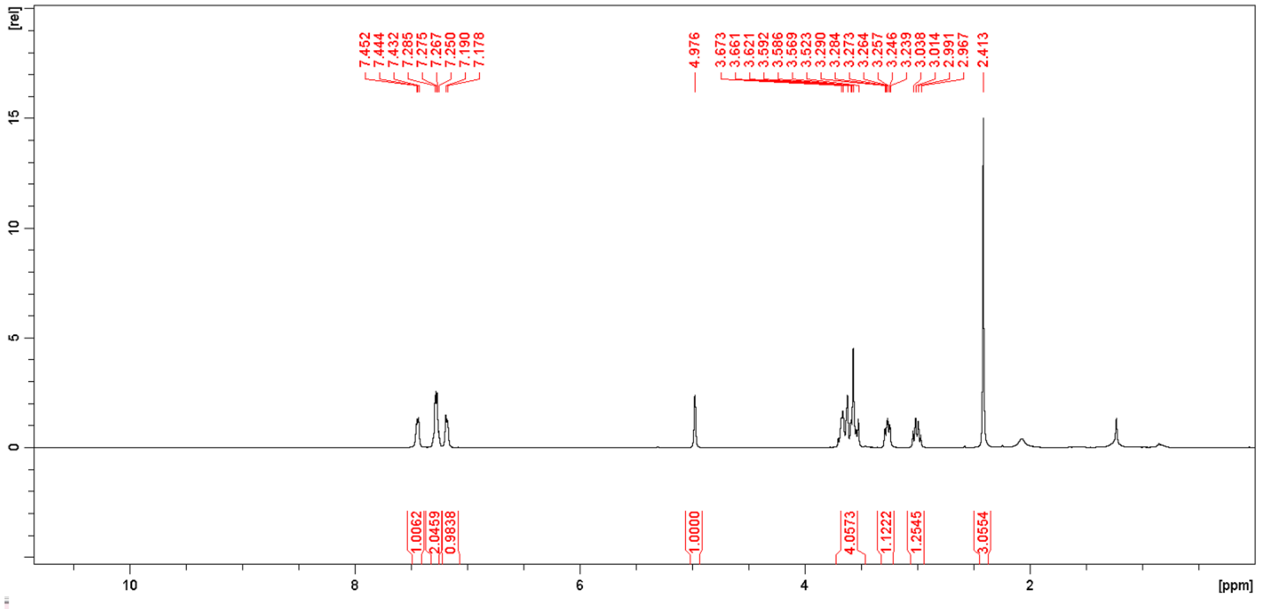

^{13}C{^{1}H} NMR (100 MHz, CDCl$_3$)

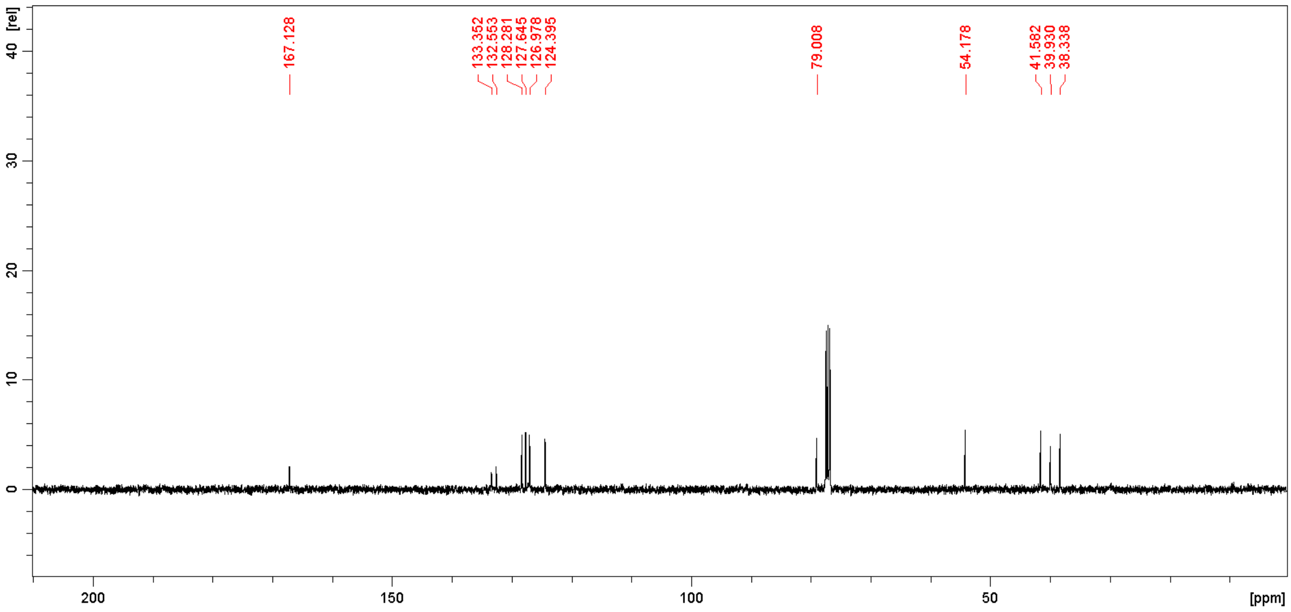

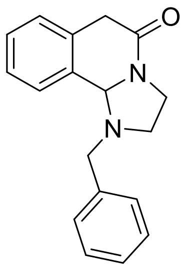

14e

^{1}H NMR (400 MHz, $CDCl_3$)

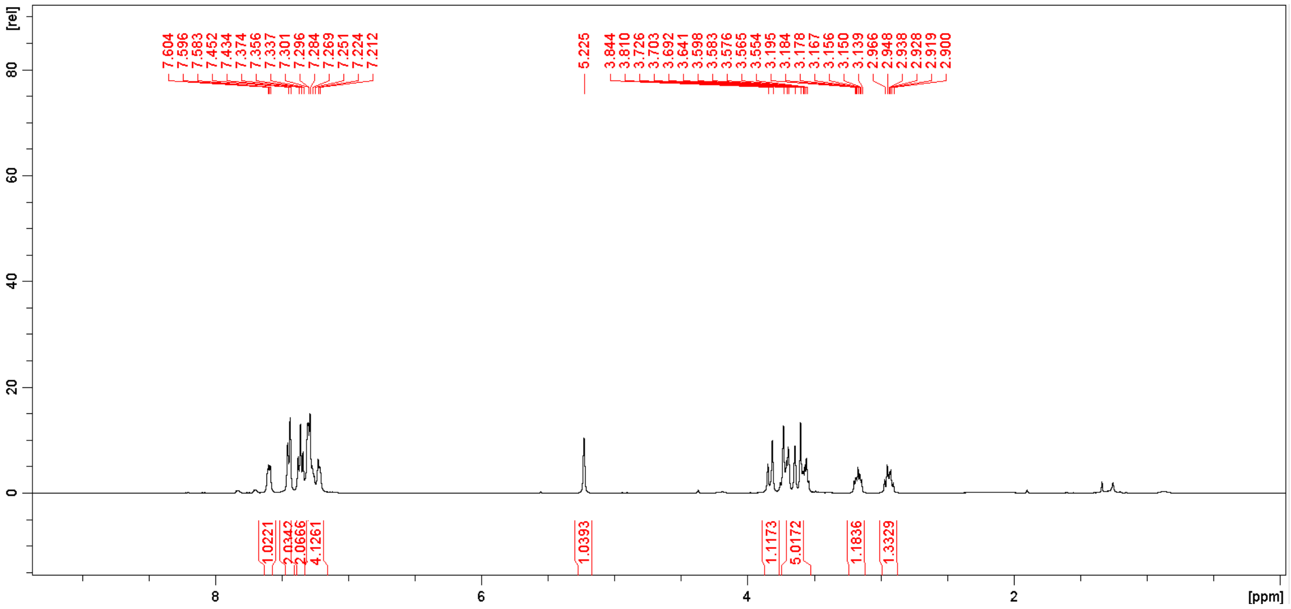

$^{13}C\{^{1}H\}$ NMR (75 MHz, $CDCl_3$)

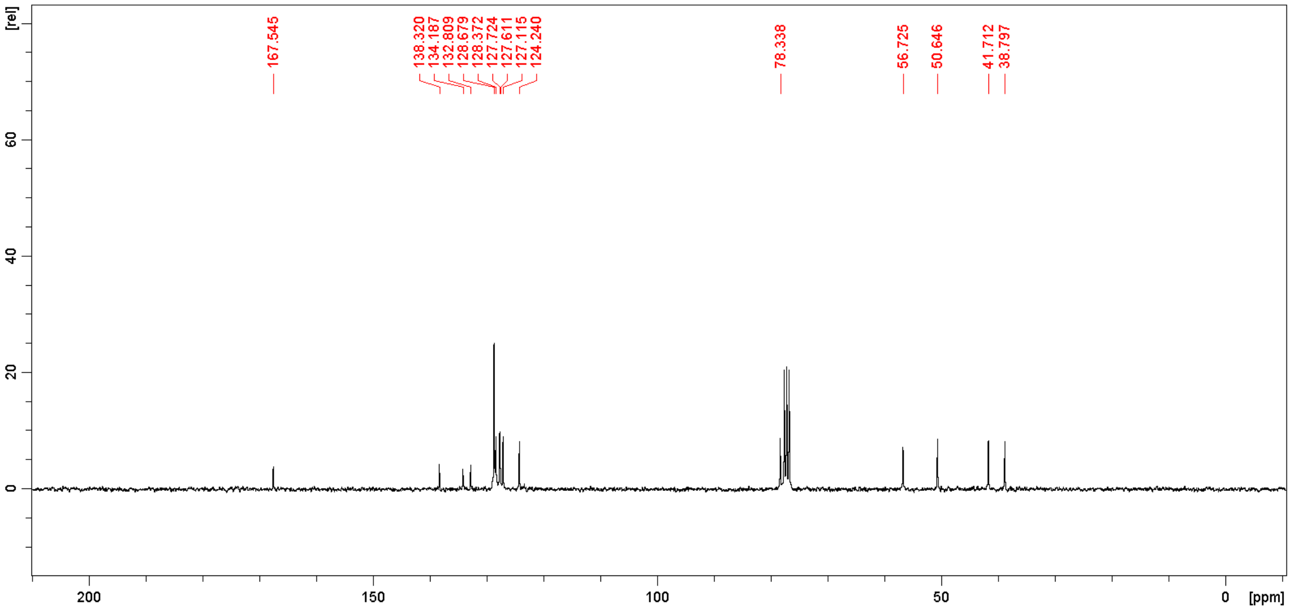

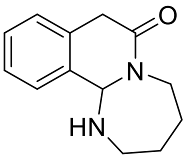

14f

^{1}H NMR (400 MHz, $CDCl_3$)

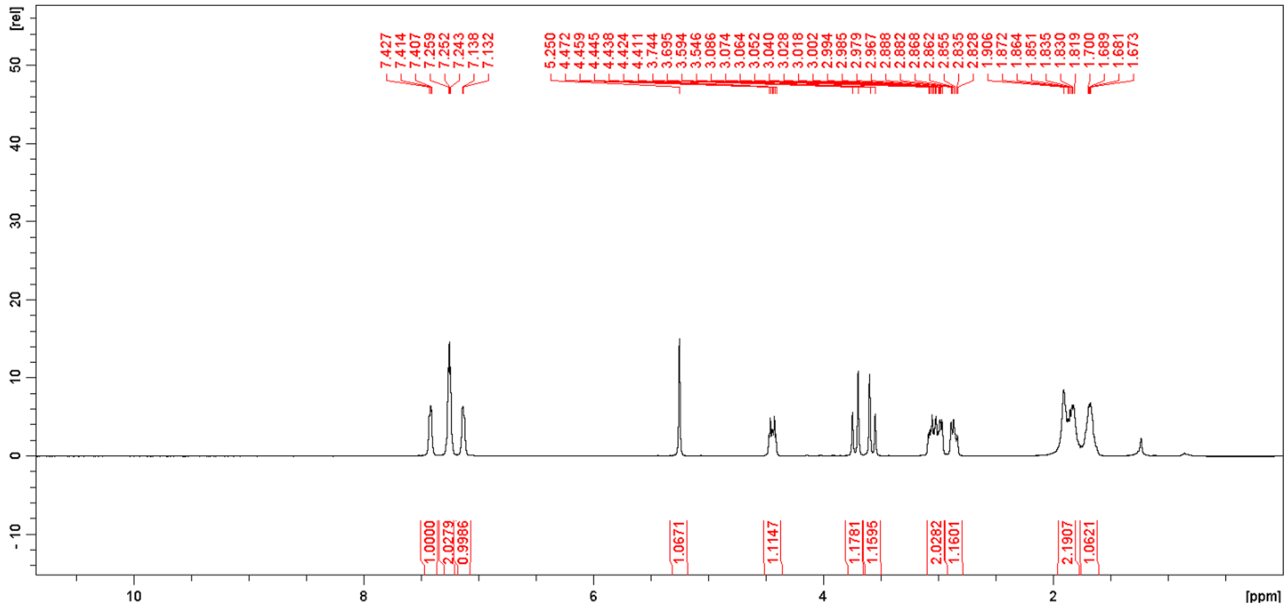

$^{13}C\{^{1}H\}$ NMR (100 MHz, $CDCl_3$)

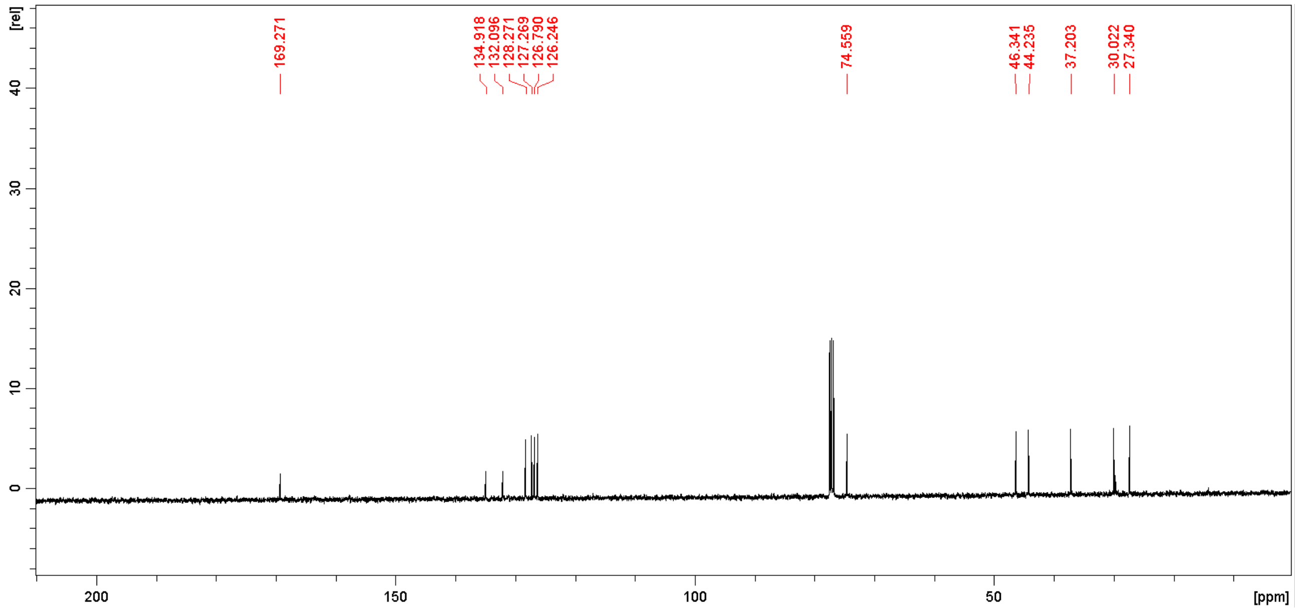

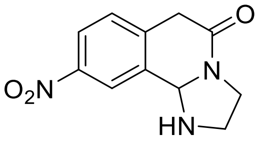

14h

^{1}H NMR (400 MHz, $CDCl_3$)

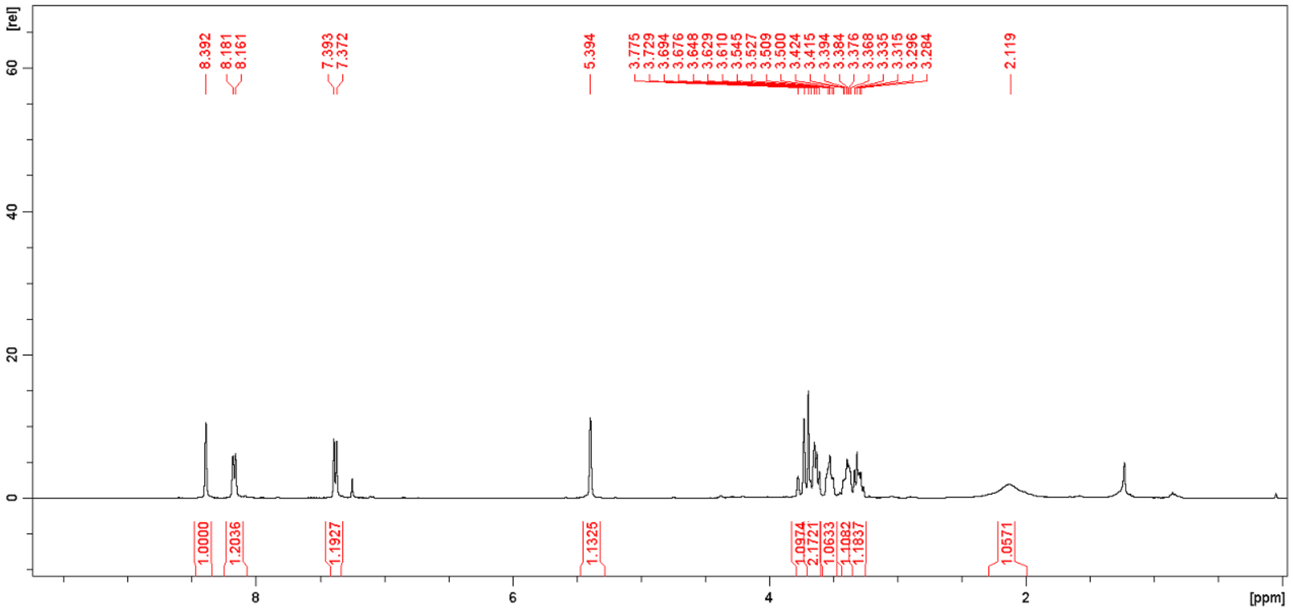

^{13}C{^{1}H} NMR (100 MHz, $CDCl_3$)

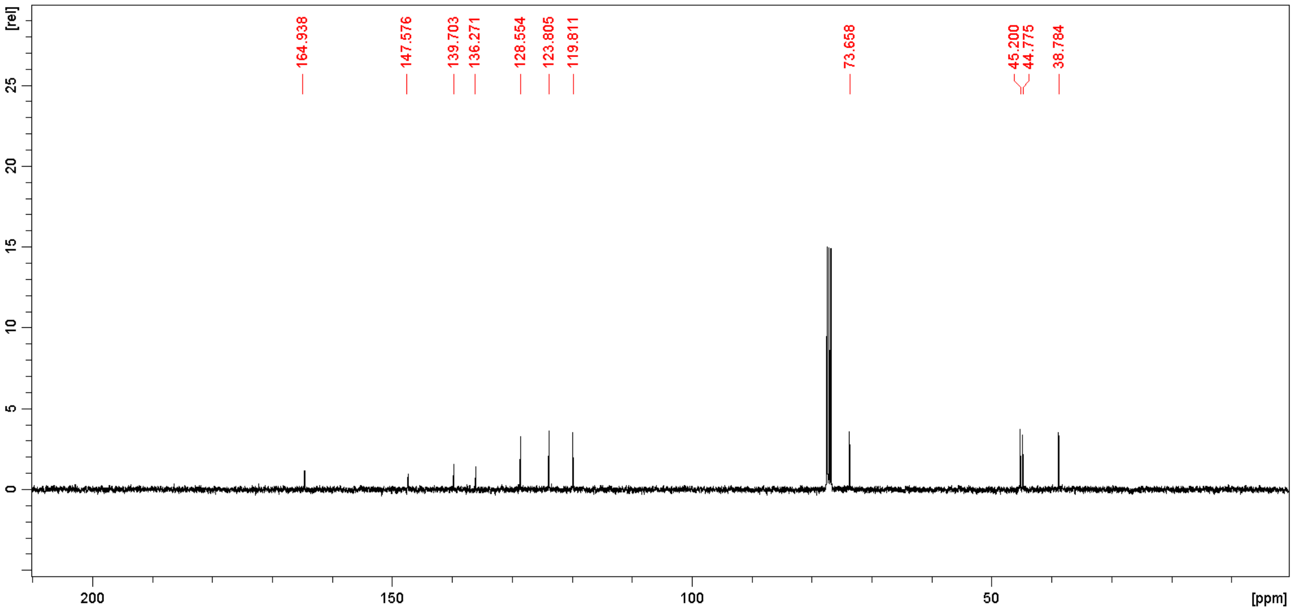

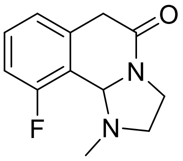

14i

^{1}H NMR (400 MHz, $CDCl_3$)

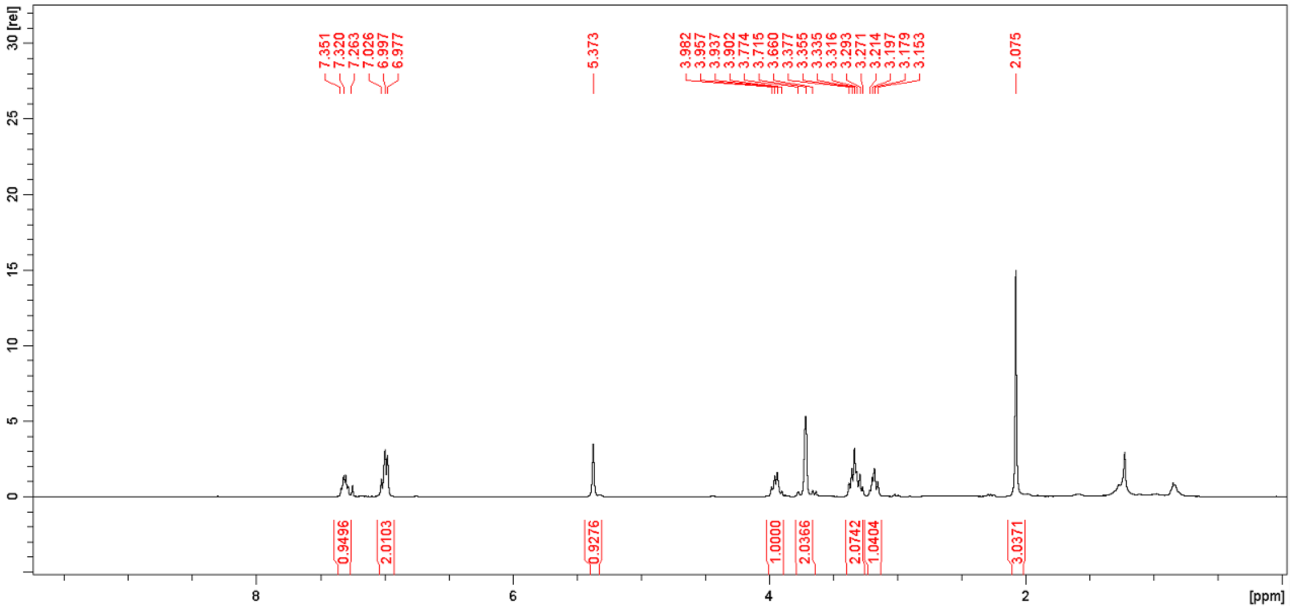

$^{13}C\{^{1}H\}$ NMR (100 MHz, $CDCl_3$)

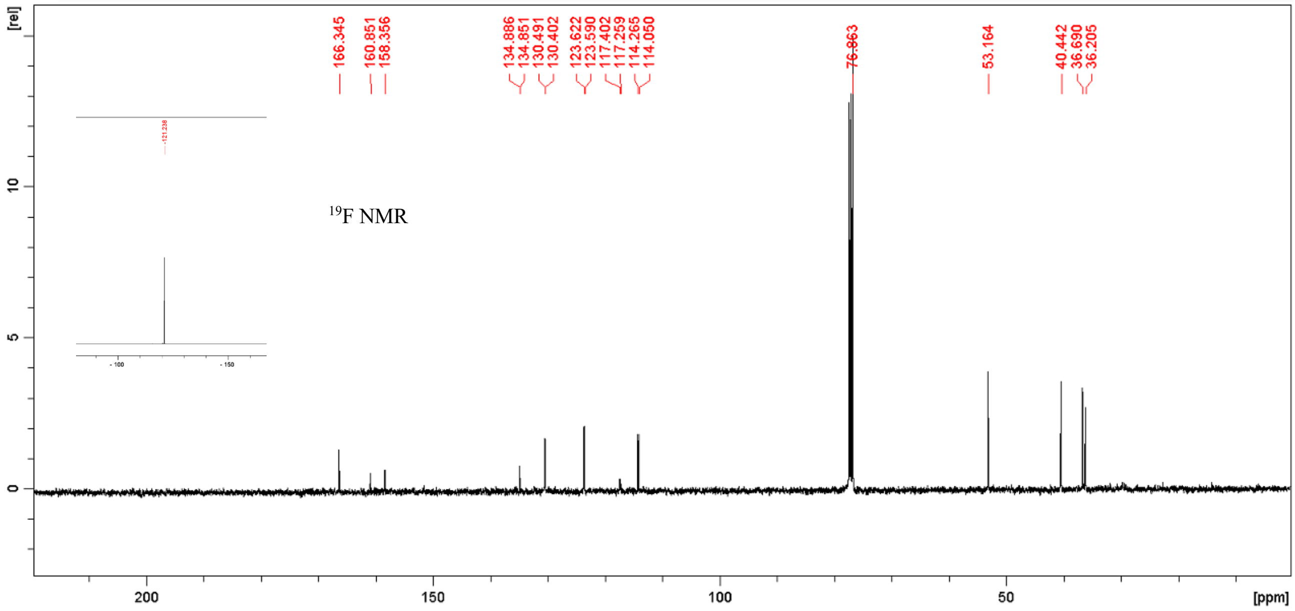

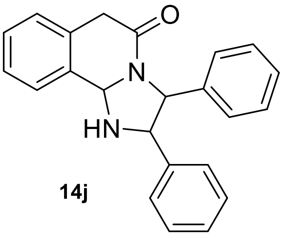

14j

^{1}H NMR (400 MHz, $CDCl_3$)

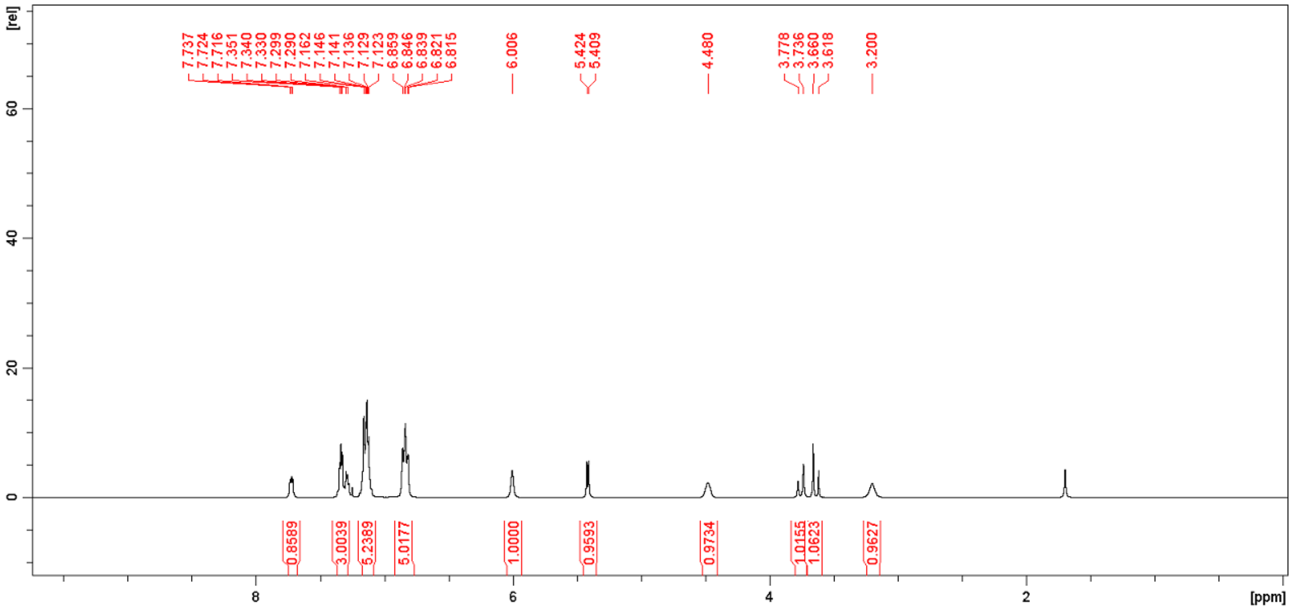

$^{13}C\{^{1}H\}$ NMR (100 MHz, $CDCl_3$)

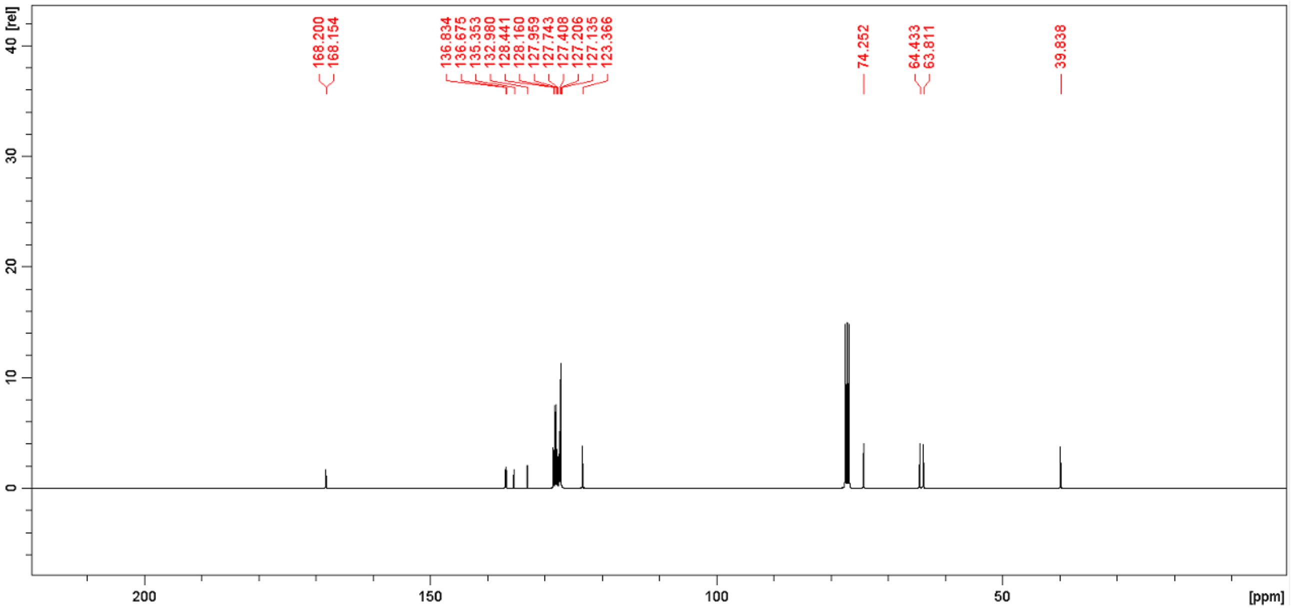

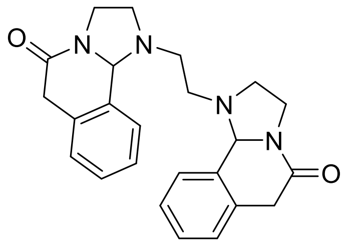

14k

^{1}H NMR (400 MHz, $CDCl_3$)

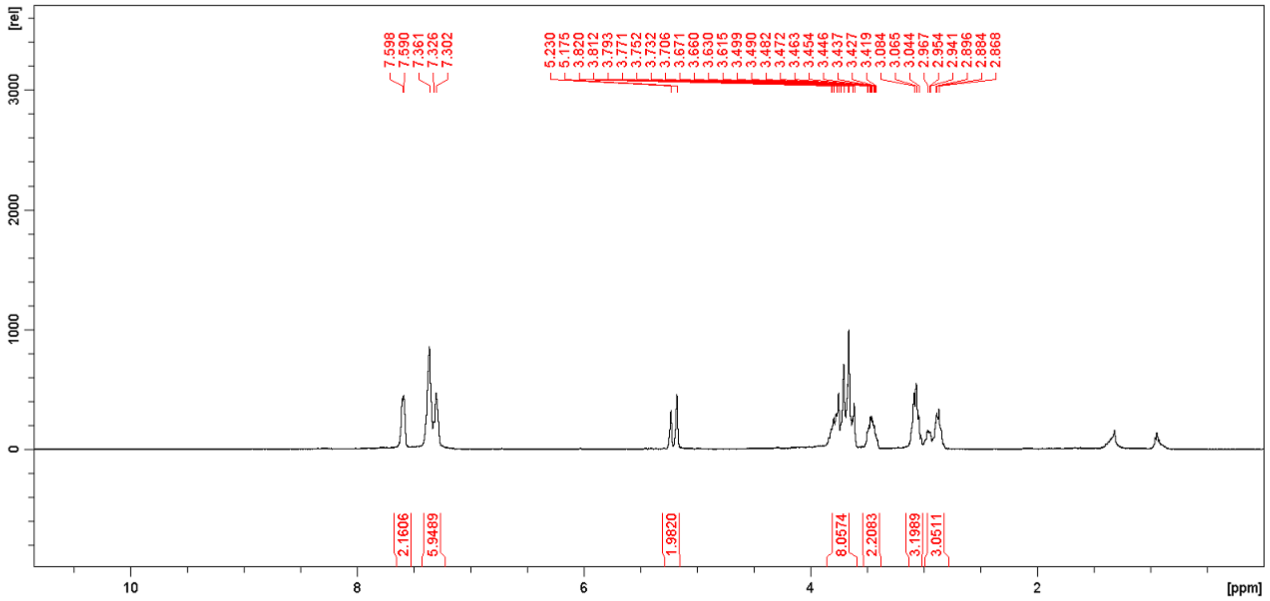

$^{13}C\{^{1}H\}$ NMR (100 MHz, $CDCl_3$)

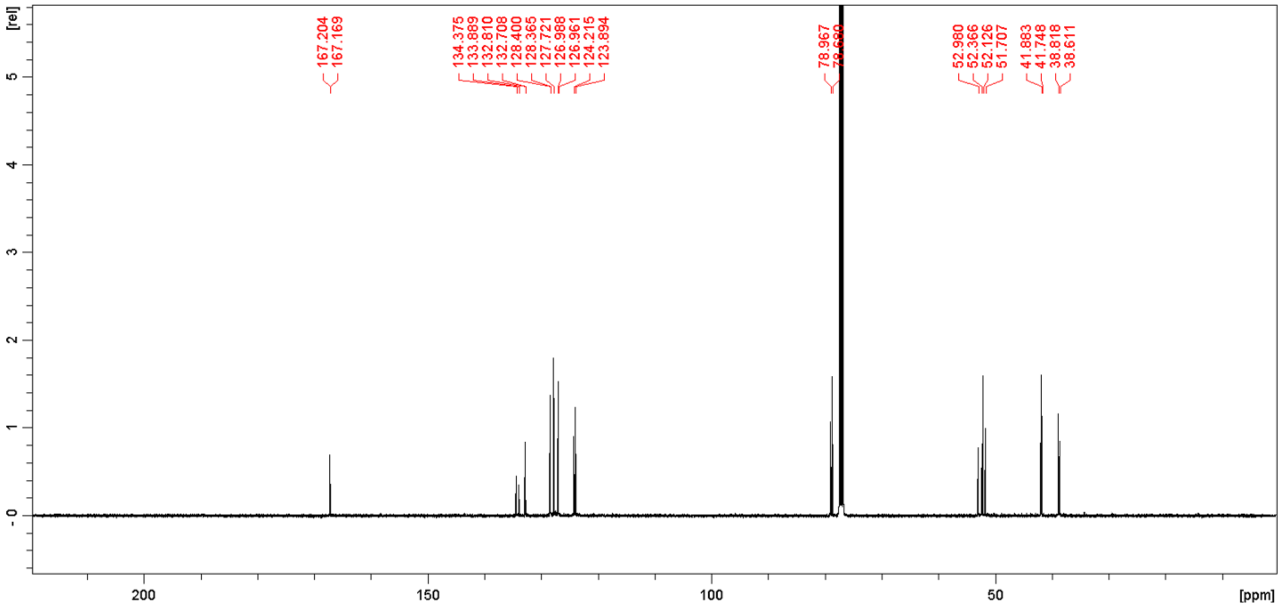

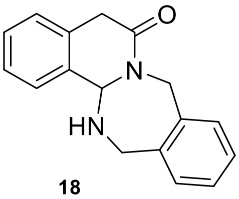

18

^{1}H NMR (400 MHz, $CDCl_3$)

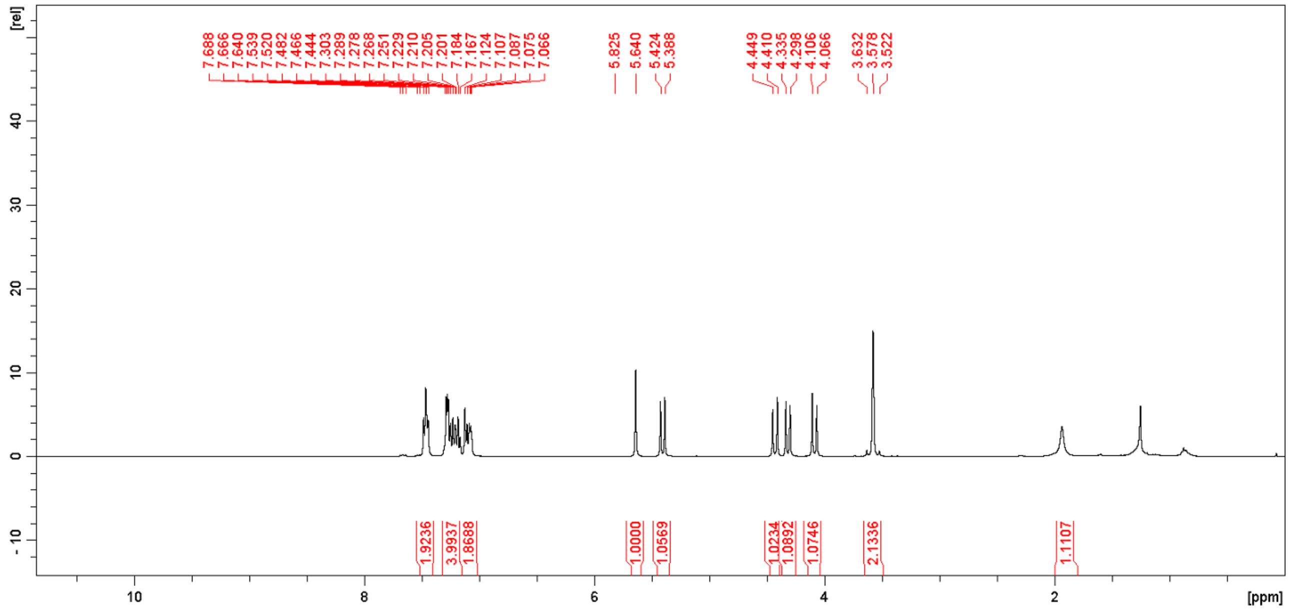

$^{13}C\{^{1}H\}$ NMR (100 MHz, $CDCl_3$)

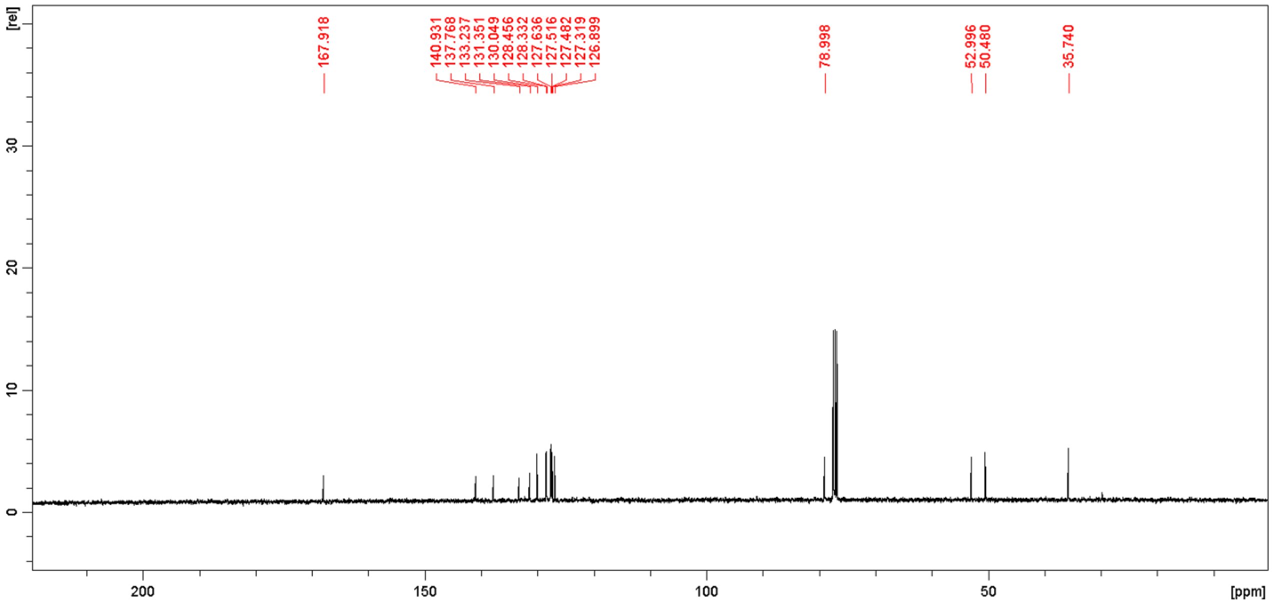

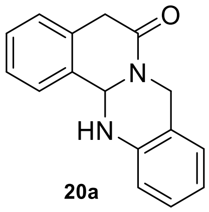

20a

^{1}H NMR (400 MHz, $CDCl_3$)

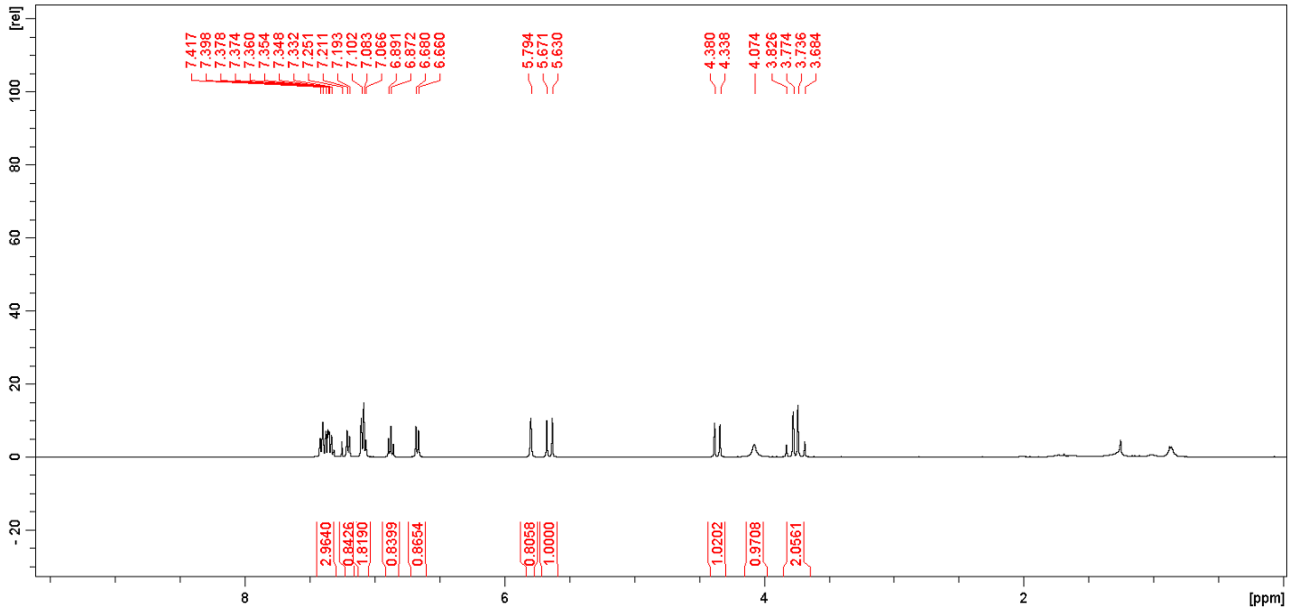

$^{13}C\{^{1}H\}$ NMR (100 MHz, $CDCl_3$)

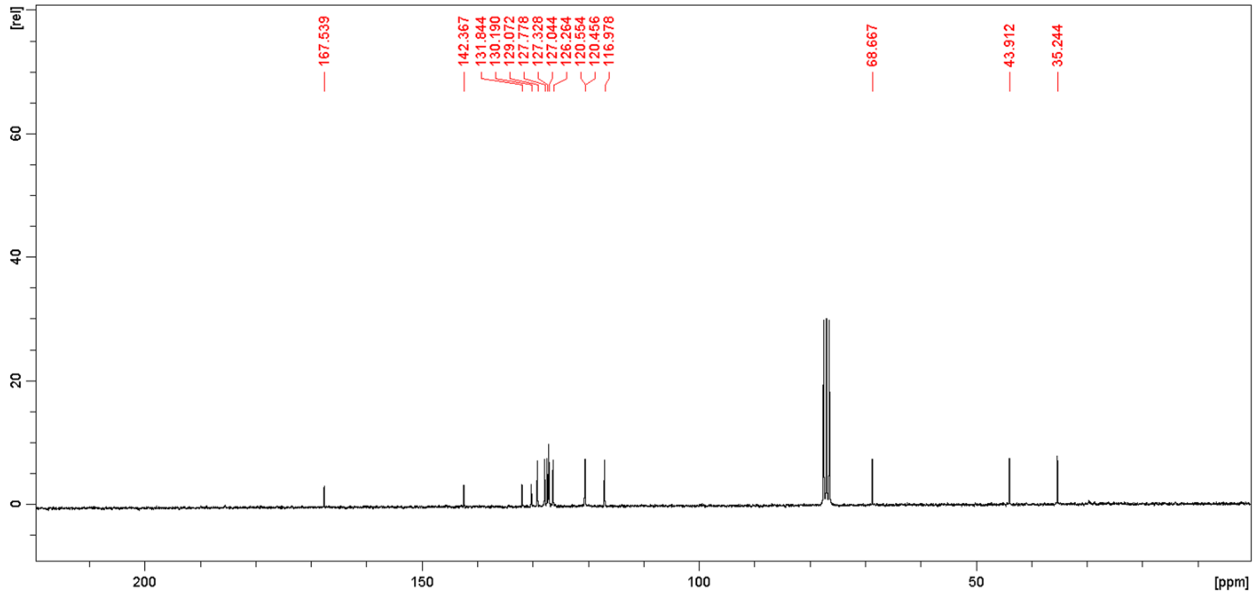

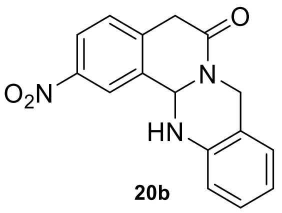

20b

^{1}H NMR (400 MHz, DMSO-d$_6$)

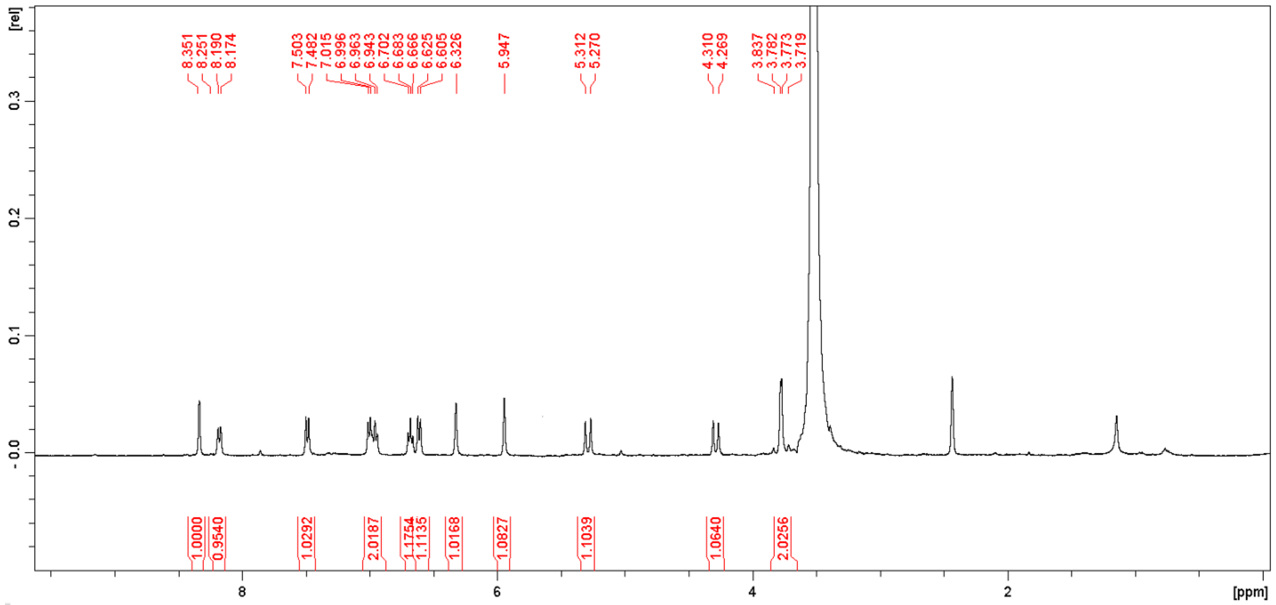

^{13}C{^{1}H} NMR (150 MHz, DMSO-d$_6$)

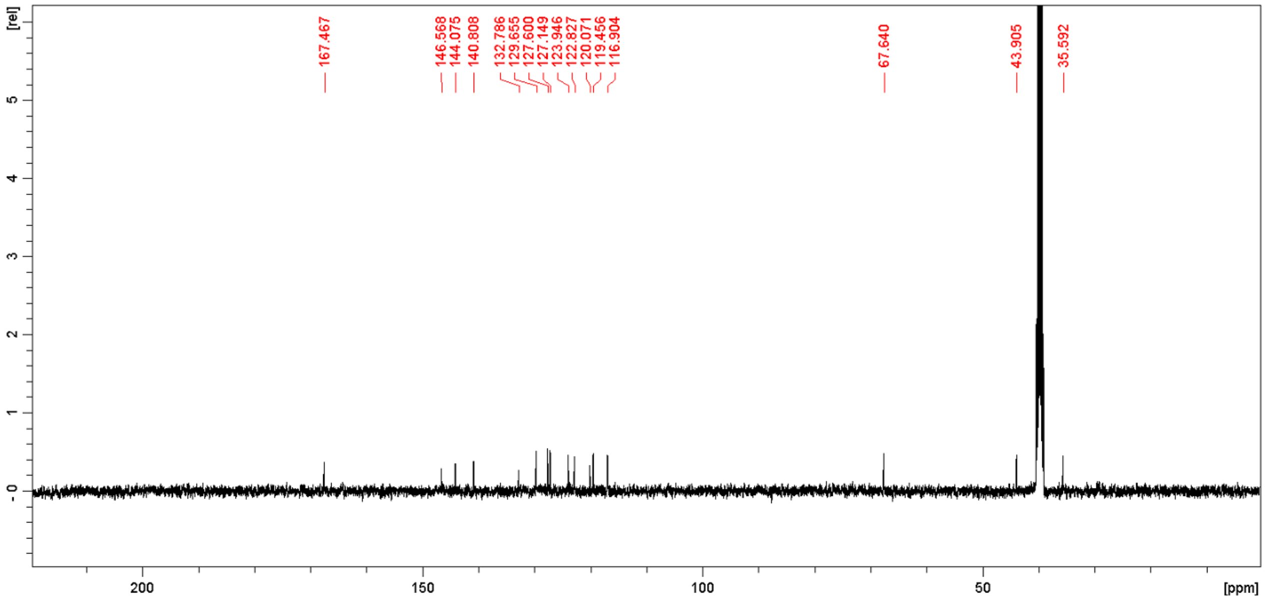

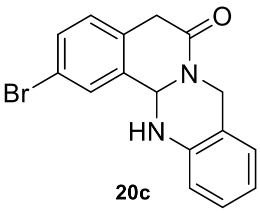

20c

^{1}H NMR (400 MHz, $CDCl_3$)

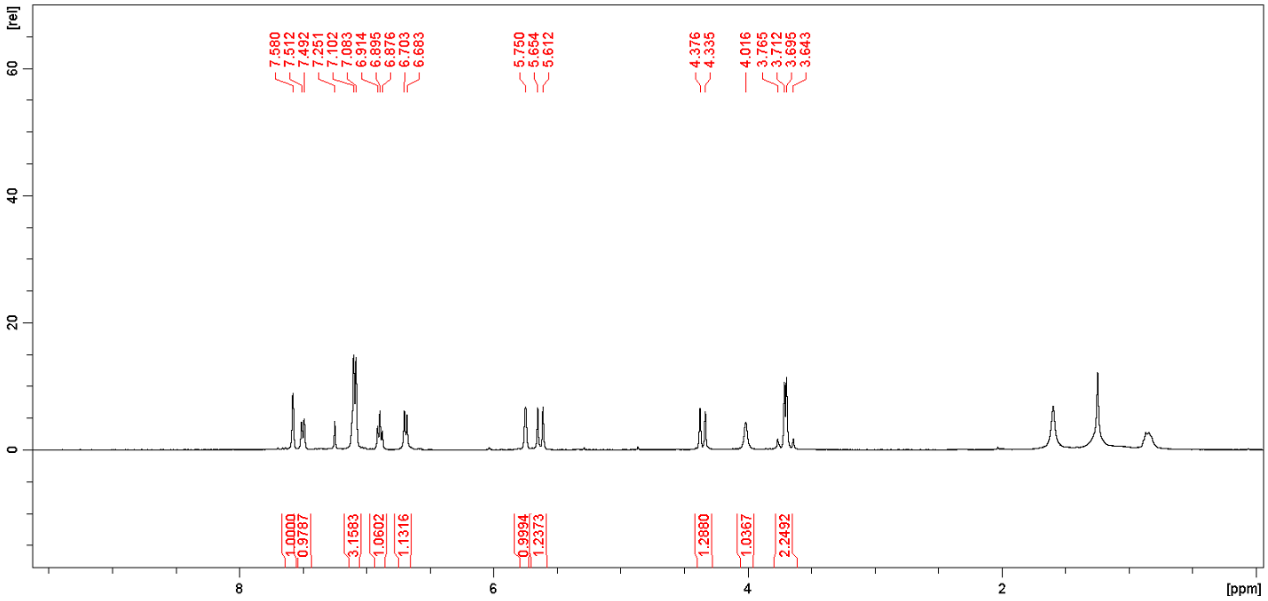

^{13}C{^{1}H} NMR (150 MHz, $CDCl_3$)

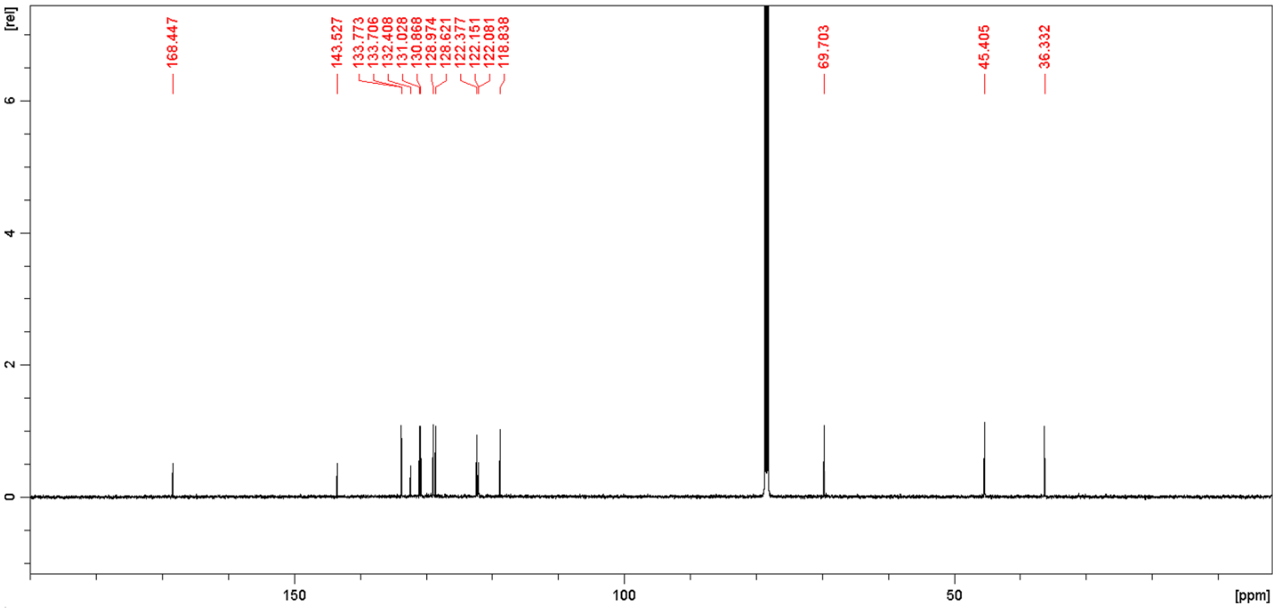

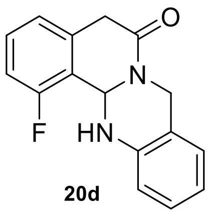

20d

^{1}H NMR (600 MHz, $CDCl_3$)

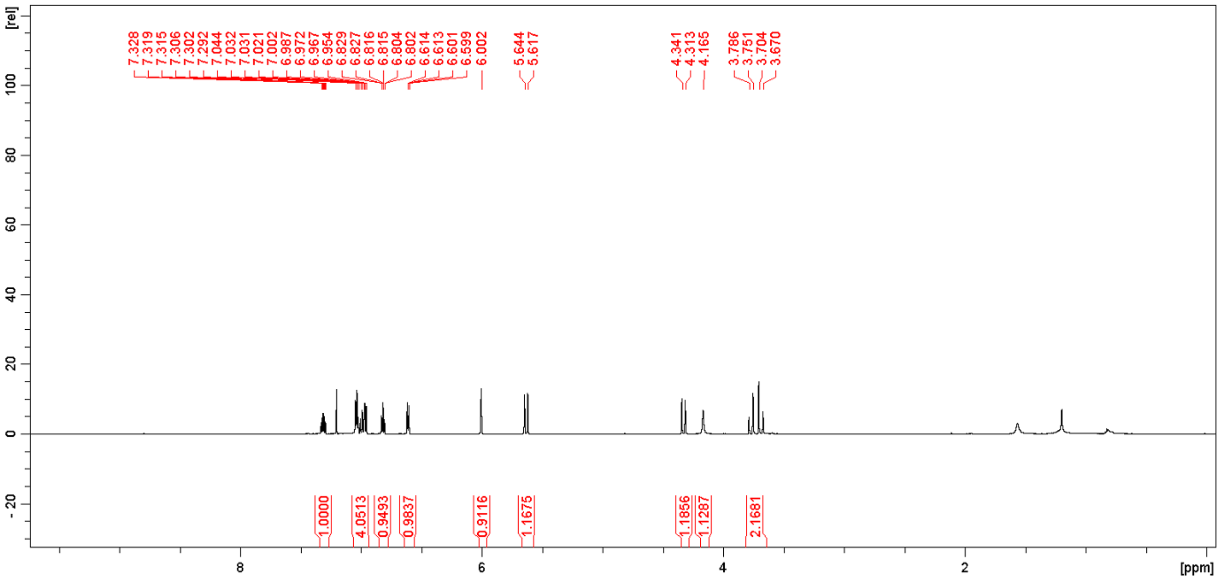

^{13}C{^{1}H} NMR (150 MHz, $CDCl_3$)

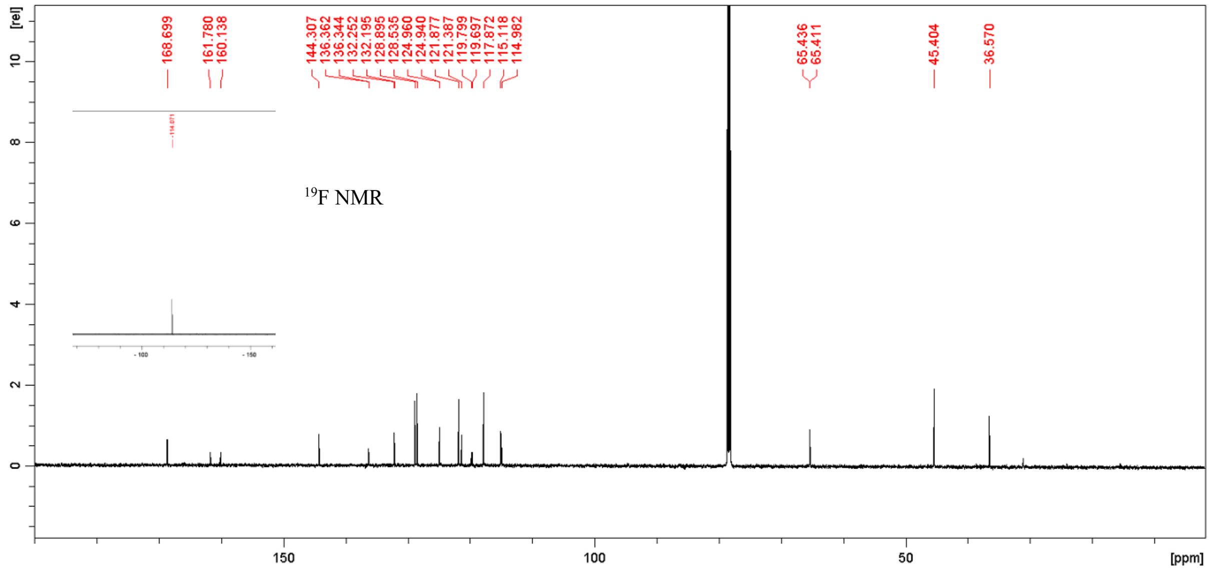

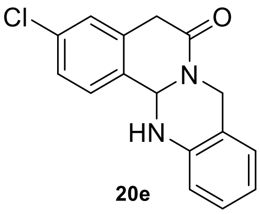

20e

^{1}H NMR (600 MHz, $CDCl_3$)

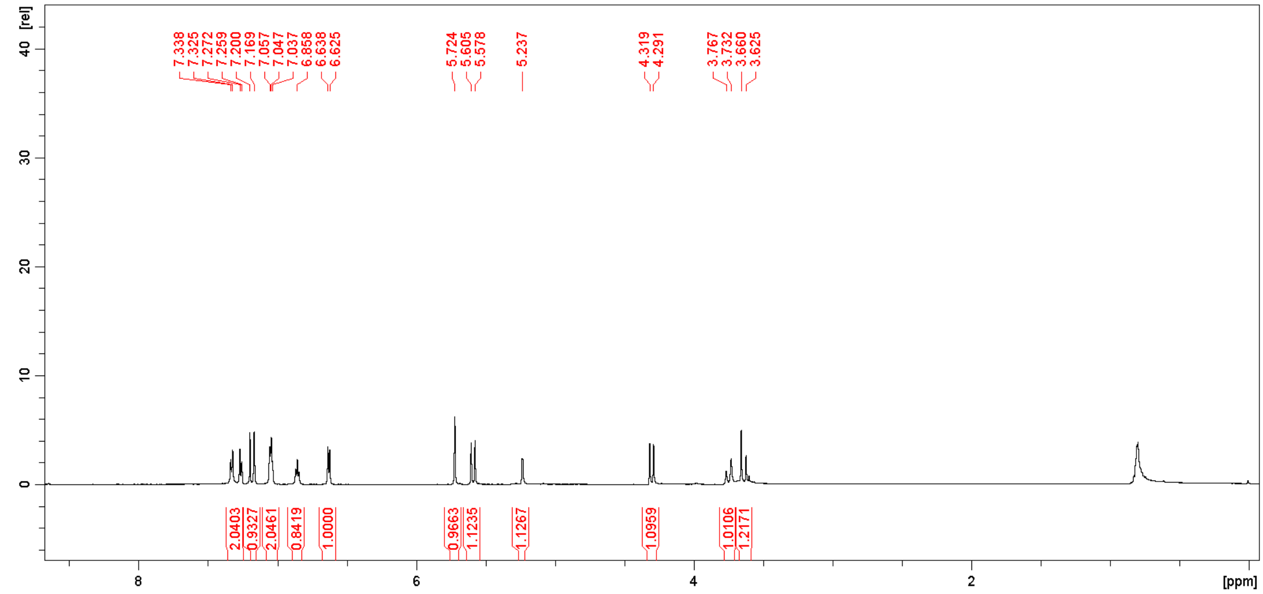

^{13}C{^{1}H} NMR (150 MHz, $CDCl_3$)

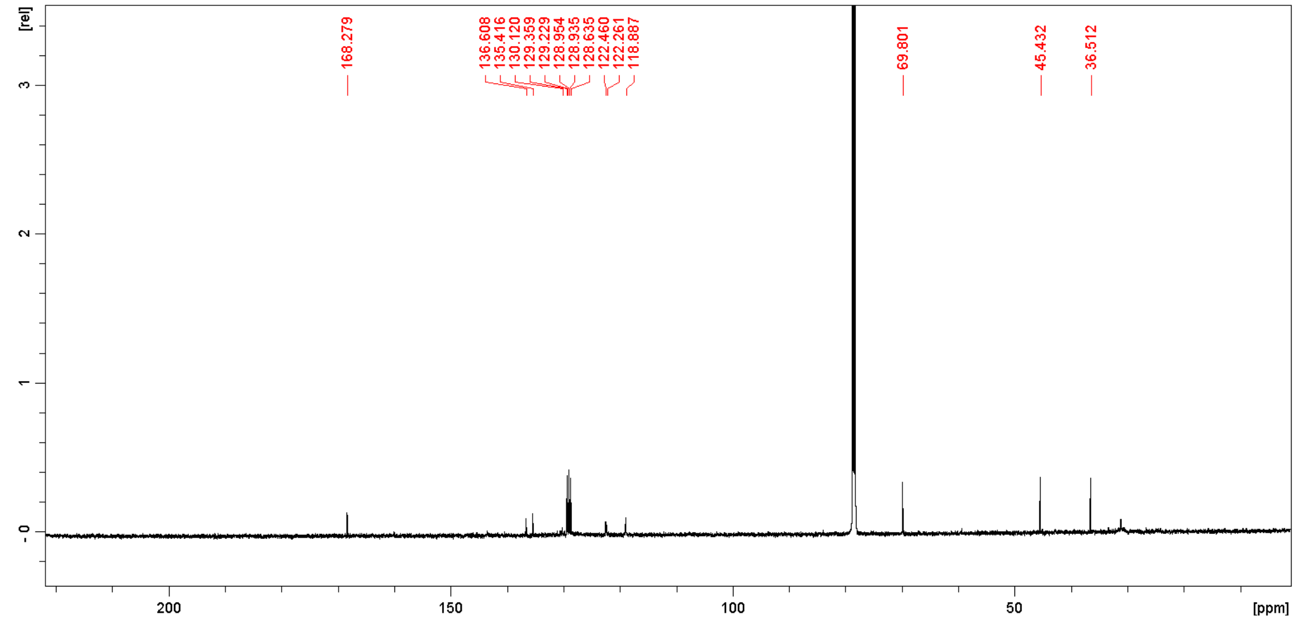

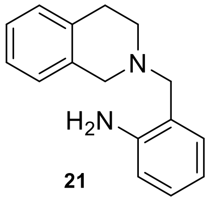

21

^{1}H NMR (400 MHz, $CDCl_3$)

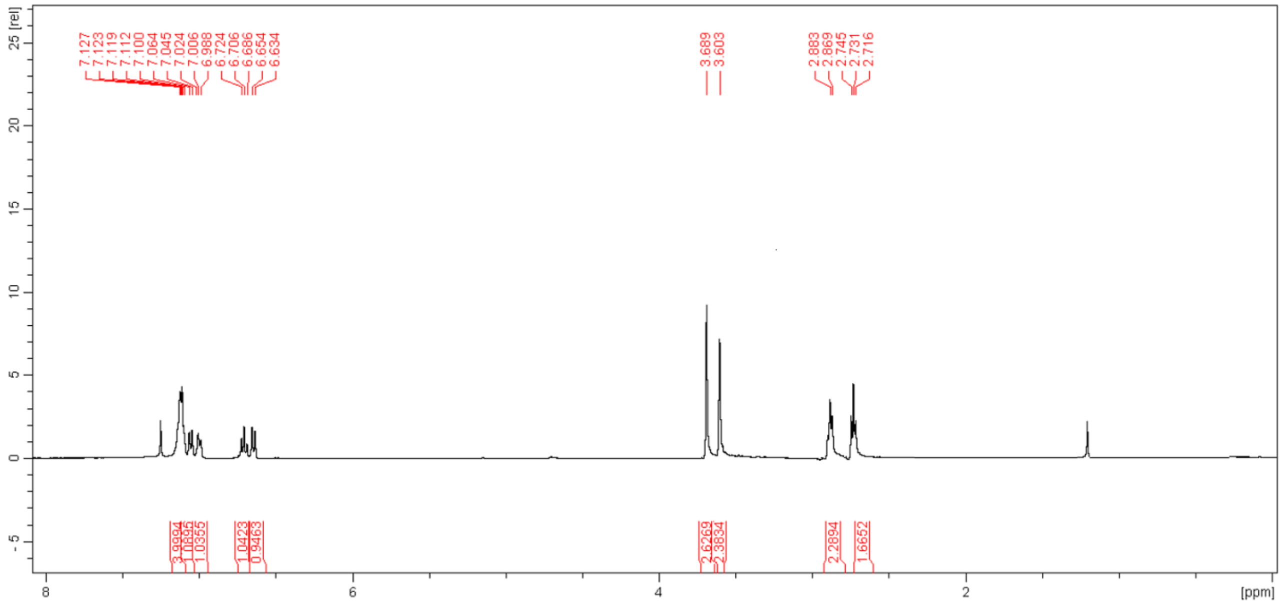

$^{13}C\{^{1}H\}$ NMR (100 MHz, $CDCl_3$)

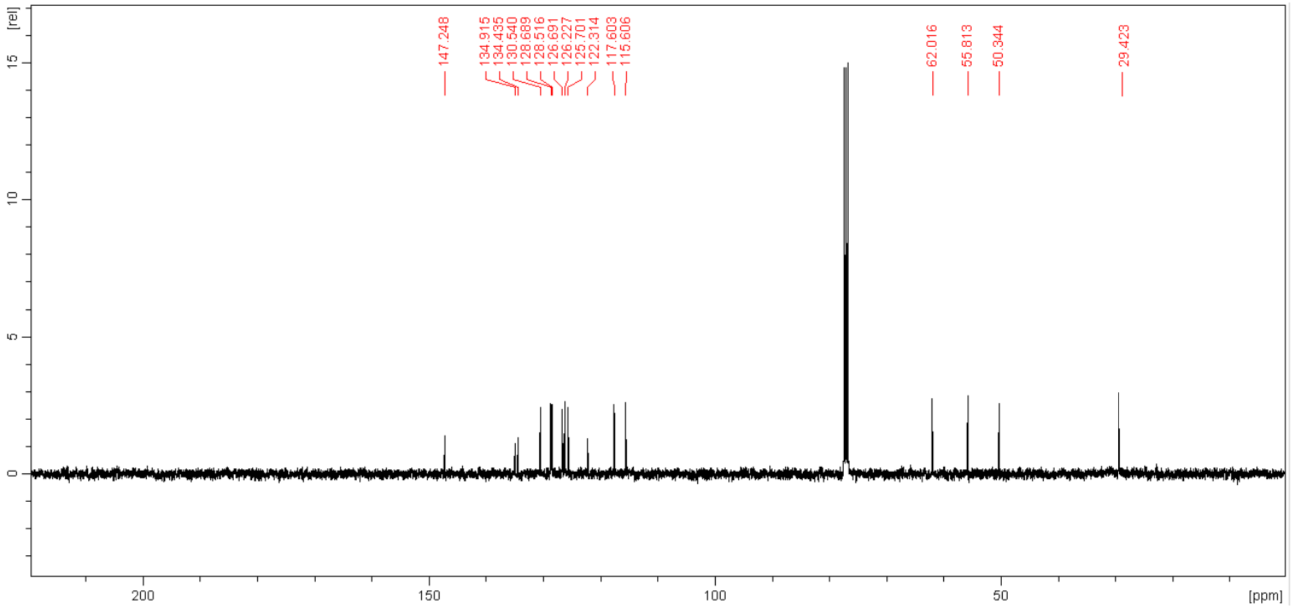

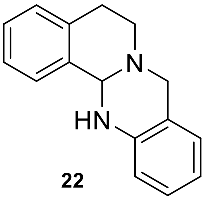

22

^1H NMR (400 MHz, $CDCl_3$)

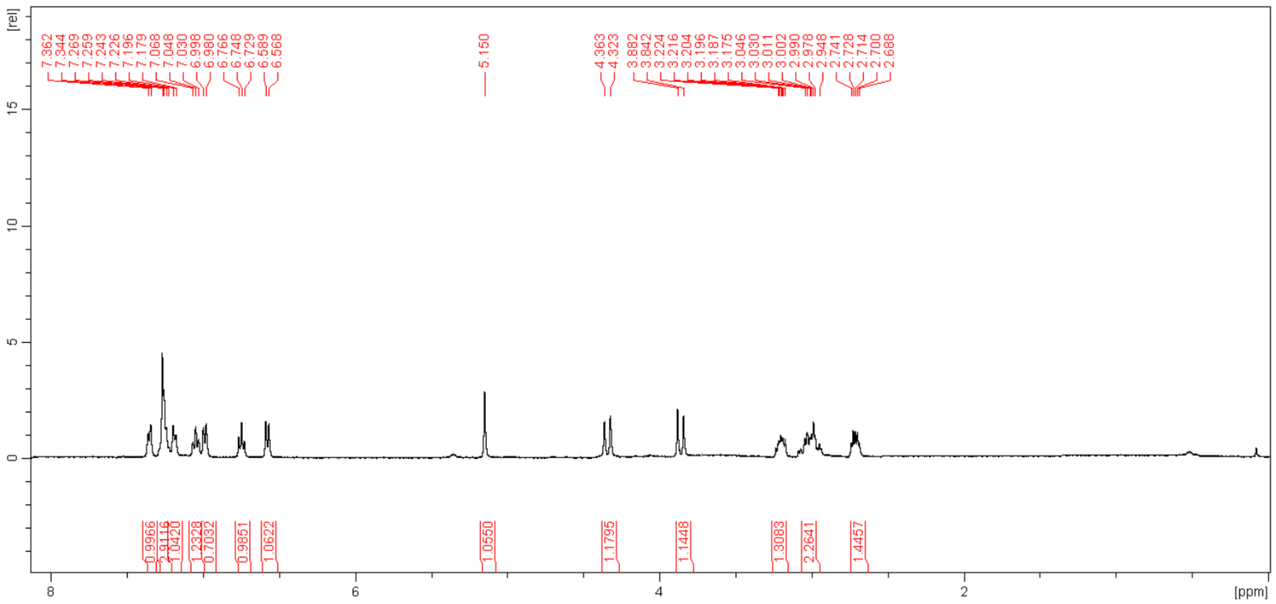

Supplement: Supplementary file 1 [file molecules-30-02702-s001.zip › molecules-3686380-supplementary.pdf]
